# Impact of focus cue presentation on perceived realism of 3-D scene structure: Implications for scene perception and for display technology

**DOI:** 10.1167/jov.24.2.13

**Published:** 2024-02-27

**Authors:** Joseph G. March, Anantha Krishnan, Rafał K. Mantiuk, Simon J. Watt

**Affiliations:** 1 Department of Computer Science and Technology University of Cambridge, UK; 2 School of Psychology and Sport Science Bangor University, UK; 3 Department of Computer Science and Technology University of Cambridge, UK; 4 School of Psychology and Sport Science Bangor University, UK

**Keywords:** focus cues, stereoscopic displays, perception, computer graphics, virtual reality

## Abstract

Stereoscopic imagery often aims to evoke three-dimensional (3-D) percepts that are accurate and realistic-looking. The “gap” between 3-D imagery and real scenes is small, but focus cues typically remain incorrect because images are displayed on a single focal plane. Research has concentrated on the resulting vergence–accommodation conflicts. Yet, incorrect focus cues may also affect the appearance of 3-D imagery. We investigated whether incorrect focus cues reduce perceived realism of 3-D structure (“depth realism”). Experiment 1 used a multiple-focal-planes display to compare depth realism with correct focus cues vs. conventional stereo presentation. The stimuli were random-dot stereograms, which isolated the role of focus cues. Depth realism was consistently lower with incorrect focus cues, providing proof-of-principle evidence that they contribute to perceptual realism. Experiments 2 and 3 examined whether focus cues play a similar role with realistic objects, presented with an almost complete set of visual cues using a high-resolution, high-dynamic-range multiple-focal-planes display. We also examined the efficacy of approximating correct focus cues via gaze-contingent depth-of-field rendering. Improvements in depth realism with correct focus cues were less clear in more realistic scenes, indicating that the role of focus cues in depth realism depends on scene content. Rendering-based approaches, if anything, reduced depth realism, which we attribute to their inability to present higher-order aspects of blur correctly. Our findings suggest future general 3-D display solutions may need to present focus cues correctly to maximise perceptual realism.

## Introduction

### Overview

Stereoscopic three-dimensional (3-D) imagery typically aims to evoke a percept of a 3-D scene that is not only quantitatively accurate, but also qualitatively realistic and natural looking. Advances in display hardware and rendering mean that for many visual display properties the “gap” between stereo 3-D images and real scenes is small (De Silva et al., 2011; Banks, Hoffman, Kim, & Wetzstein, 2016; Zhong et al., 2021). Yet focus cues—the stimulus to the eye’s focusing response (accommodation) and retinal blur gradient—typically remain incorrect in stereo 3-D. Most research has concentrated on one specific consequence of this, the mismatch or conflict between vergence and accommodation stimuli, which has been shown to cause discomfort and fatigue (Hoffman, Girshick, Akeley, & Banks, 2008; Shibata, Kim, Hoffman, & Banks, 2011) and reduced stereo performance, including distortions in perceived depth, reduced stereoacuity, and increased time required for stereoscopic fusion (Akeley, Watt, Girshick, & Banks, 2004; Watt, Akeley, Ernst, & Banks, 2005a; Watt, Akeley, Girshick, & Banks, 2005b; Hoffman et al., 2008). Incorrect focus cues might be expected to have a wider range of consequences, however, including reducing perceived realism of the 3-D space in a scene (referred to as *depth realism*; Hibbard, Haines, & Hornsey, 2017). Depth realism relates to the subjective sense of how tangible, solid and real separation in depth appears in stereo 3-D imagery (Vishwanath & Hibbard, 2013; Hibbard et al., 2017). We investigated whether incorrect focus cues reduce depth realism in a two-part study. The first part (Experiment 1) was a proof-of-principle test of whether focus cues affect depth realism, using classical reduced-cue scenes designed to isolate their role. The second part (Experiments 2 and 3) examined whether focus cues affect depth realism “in practice,” in more complex cue-rich scenes more representative of typical computer graphics applications. This part also examined the efficacy of several rendering-based techniques that aim to present focus cues to varying degrees of correctness (Duchowski et al., 2014; Vaidyanathan, Munkberg, Clarberg, & Salvi, 2015; Cholewiak, Love, Srinivasan, Ng, & Banks, 2017). Approximating correct focus cues is technically challenging, increases the cost and complexity of display systems, and can require trading-off other desirable aspects of image quality (Wetzstein, Lanman, Hirsch, & Raskar, 2012; Lanman & Luebke, 2013; Huang, Chen, & Wetzstein, 2015; Chang, Kumar, & Sankaranarayanan, 2018; Javidi et al., 2021; Chakravarthula, Tseng, Fuchs, & Heide, 2022). It is therefore important to evaluate the benefits that are conferred. Our results not only contribute to fundamental understanding of the role of focus cues in scene perception, but also inform the development of future display technologies and rendering techniques, by helping characterise the criteria they must meet to present realistic content.

### Signals to unrealism from incorrect focus cues

Conventional stereoscopic 3-D display systems (including most commercially available virtual reality [VR] headsets, at the time of writing) present images on a single, fixed display surface. This means that focal distance to points in the scene is consistent with the display surface rather than with the depicted 3-D scene. (For a detailed breakdown of different aspects of the stimulus to accommodation and retinal blur in displays, and the properties needed to present them correctly, see *Display requirements for correct focus cues*.)

Incorrect focus cues might be expected to reduce depth realism for several reasons. One reason is that they provide inaccurate depth information, which could result in discernible conflicts between different depth cues, which do not arise in natural viewing. Although focus cues are often thought of as weak depth cues, they can in fact play a significant role in depth perception under some circumstances. Varying accommodation distance has been shown to affect perceived slant, for example, by altering the estimate of distance used to interpret binocular disparities (Watt et al., 2005a; Hoffman et al., 2008). Blur can also contribute significantly to depth perception at occlusion edges (Marshall, Burbeck, Ariely, Rolland, & Martin, 1996; Mather, 1997; Nguyen, Howard, & Allison, 2005), and for scene points nearer and farther than fixation (Held, Cooper, & Banks, 2012). Moreover, changes to the “global” blur gradient in a scene can dramatically affect perception of its overall spatial scale, as seen in the phenomenon of tilt-shift miniaturization, in which increasing the “global” blur gradient causes natural scenes to resemble scale models (Held, Cooper, O’Brien, & Banks, 2010; Vishwanath & Blaser, 2010).

Incorrect focus cues could also diminish the qualitative sense of three-dimensionality present when viewing the real world. Viewing real 3-D scenes results in a qualitatively distinct sense of vivid, tangible 3-D structure, which is typically not present when viewing two-dimensional (2-D) paintings or even photographs (Vishwanath & Hibbard, 2013). It has long been recognised that this sense of “real depth”—often referred to as stereopsis—is induced by viewing 3-D scenes defined solely by binocular disparity or motion parallax (Wallach & O’Connell, 1953; Rogers & Graham, 1979; Rogers & Graham, 1982). More recent work suggests that depth-dependent blur added to images to simulate effects of defocus can also induce the sense of real depth associated with stereopsis (Vishwanath & Hibbard, 2013). This implies that incorrect blur in stereo 3-D might diminish the sense of depth realism compared with when focus cues are correct.

Incorrect focus cues could also reduce realism not because of changes related to depth perception per se, but because other aspects of the appearance of stereo 3-D scenes do not match our experience of viewing the real-world. For example, in natural viewing we are used to fixated objects appearing sharp and objects nearer and farther than fixation appearing blurred. Moreover, these patterns of blur change predictably as we look around a scene and accommodate nearer and farther. Conventional stereo 3-D displays do not reproduce these aspects of image appearance. Indeed, large vergence–accommodation conflicts can result in inaccurate accommodation responses (Fincham & Walton, 1957; Martens & Ogle, 1959), causing even fixated objects to appear blurred. We are presumably highly accustomed to the static and dynamic aspects of natural scene appearance through experience of daily life, and a mismatch to expectations could diminish perceived realism.

Finally, the unnatural nature of stereo 3-D scenes could also be signalled by oculomotor signals. Viewers may detect that they are not making the normal, expected pattern of accommodation responses as they look around the scene, and/or that greater than normal motoric effort is required to overcome the coupling between vergence and accommodation responses (Fincham & Walton, 1957; Martens & Ogle, 1959). Again, by violating long-established expectations based on viewing real scenes, the pattern of motor output when viewing stereo 3-D scenes may feel unnatural, resulting in reduced perceived realism of 3-D scene structure.

Previous work on depth realism in stereo 3-D provides some hints that incorrect focus may reduce perceived realism. Observers in a study by Hibbard et al. (2017) viewed two pairs of random-dot-defined planes that were overlapping (i.e., were in the same visual direction), but separated in stereoscopic depth by varying amounts, and presented using conventional stereo 3-D (i.e., with incorrect focus cues). In separate sessions, observers made two-alternative, forced-choice judgments of which pair i) had the largest depth separation, and ii) had the highest depth realism (again, defined as how tangible, solid and real separation in depth appeared). All possible combinations of depth separations were presented and Thurstonian scaling was used to assess perceived depth magnitude, and depth realism. As expected, the magnitude of perceived depth separation increased with increasing disparity-specified depth (levelling off around the limit of binocular fusion). Depth realism showed a different pattern, however. Realism was highest at small depth separations and reduced systematically as separation-in-depth increased. These results indicate that magnitude and realism are dissociable aspects of perceived depth for observers. Yet they are also puzzling, in that it seems implausible that the real world would appear less realistic as separation between objects increases. Hibbard et al. (2017) proposed that more precise depth estimates may appear more realistic, noting that stereoscopic depth is less precisely encoded as depth separation increases (see their Experiment 2). However, reduced precision with increased depth is also a feature of real scenes: depth information from most cues becomes less precise with increasing depth, largely for geometrical reasons (Hillis, Watt, Landy, & Banks, 2004; Keefe, Hibbard, & Watt, 2011). An alternative explanation is that Hibbard et al.'s (2017) data reflect the influence of incorrect focus cues on depth realism. Because stimuli were presented on a single display surface, the magnitude of the error in focus-cue presentation (i.e., the mismatch between depth separation specified by focus cues and by disparity) increased as depth separation increased. It is possible that the decreasing depth realism reflected this increasing focus-cue error. Consistent with this explanation, the roll-off in depth realism observed by Hibbard et al. (2017) occurred at a disparity-specified separation corresponding to between  0.15 and 0.3 diopters (D), which is similar to the functional depth-of-focus of the eye (Campbell, 1957; Charman & Whitefoot, 1977; Green, Powers, & Banks, 1980). Thus, depth realism began to decrease at the point at which relative blur in the non-fixated stimulus plane would have become detectable in real-world viewing (Nguyen et al., 2005; Wang & Ciuffreda, 2005; Watson & Ahumada, 2011; Sebastian, Burge, & Geisler, 2015). Depth realism may not depend on separation-in-depth when focus cues (and other available cues) are presented correctly.

March et al. (2022) found that a (fixed) depth separation between two rendered objects was judged more realistic when correct focus were approximated using a (high-dynamic-range) multiple-focal-planes stereo display. This study did not vary depth separation, however, and confounded presentation of near-correct focus cues with an increase in spatial resolution of the farther displayed image. In other related work, Zhong et al. (2021) used the same display, in conjunction with the capability of switching between viewing displayed imagery and an equivalent physical scene, to explore whether it was possible to create 3-D stereo imagery that could not be discriminated from the real world. Using an oddity task (three-alternative, forced-choice) they created conditions in which observers found it difficult to discriminate real objects from displayed imagery (although performance was still slightly above chance). This finding indicates that high levels of perceptual realism are achievable in a display when all available cues, including focus cues, are closely approximated. This work did not isolate the contribution of focus cues to perceptual realism, however. It remains to be determined whether presenting focus correctly, while holding all other factors constant, results in increased depth realism.

### Display requirements for correct focus cues

The light at the eye that results from natural viewing is well-approximated by a four-dimensional (4-D) light field (Levoy & Hanrahan, 1996; Akeley, 2012). A display that can reproduce this would present focus cues correctly. Holographic (Chakravarthula et al., 2022) and light-field displays (Huang et al., 2015) are the most advanced attempts to reproduce the 4-D light field, but various technical problems have so far resulted in limited resolution, and a small field of view, restricting image quality. Instead of reproducing a full 4-D light field, various (hardware- and rendering-based) approaches have been proposed that manipulate some aspects of focus cues but not others, and/or present focus cues to varying degrees of correctness, often at the cost of additional technical complexity.

The scene points that are nearer or farther than the distance the eye is currently focused at cause natural blur. The “first-order” effect is defocus blur, where higher spatial frequencies are increasingly attenuated in the retinal image, causing fine details to be less visible (Charman & Jennings, 1976). There are also “second-order” aspects of blur, however, which can affect perception and accommodation control. These effects are challenging to replicate via display systems because they depend on dynamic aspects of accommodation, and on individual eye optics. First, the eye continuously makes small microfluctuations in accommodation, resulting in changes in retinal contrast that depend on the amount of defocus (Campbell, Robson, & Westheimer, 1959; Charman & Heron, 1988; MacKenzie, Hoffman, & Watt, 2010). Second, viewing spectrally broadband scenes results in colour fringes caused by longitudinal chromatic aberration (Kruger, Mathews, Aggarwala, & Sanchez, 1993; Cholewiak et al., 2017). Third, the different imperfections in the optics of individual eyes result in unique higher-order aberrations in the retinal image (Artal et al., 2004). All three of these optical effects are thought to contribute to accommodation control by signalling the sign of defocus error (i.e., whether the eye is focused too near or too far) (Fincham, 1951; Campbell & Westheimer, 1959; Charman & Tucker, 1978; Kotulak & Schor, 1986; Kruger et al., 1993; Aggarwala, Kruger, Mathews, & Kruger, 1995; Lee, Stark, Cohen, & Kruger, 1999; Fernández & Artal, 2005; Chen, Kruger, Hofer, Singer, & Williams, 2006; Chin, Hampson, & Mallen, 2009; MacKenzie et al., 2010; Cholewiak et al., 2017). There is also evidence of perceptual sensitivity to these effects. For example, chromatic aberration has been shown to be sufficient for discriminating the sign of a step in depth (Nguyen et al., 2005) and to make monocularly viewed scenes appear more realistic (Cholewiak et al., 2017). People are also sensitive to whether they are viewing a scene with their own individual pattern of higher-order aberrations or an altered version of them (Artal et al., 2004; Sawides et al., 2012). More generally, there is evidence that blur discrimination is improved for natural blur compared to simple Gaussian blur applied to the source image (Sebastian et al., 2015). Second-order aspects of blur may therefore need to be presented correctly if depth realism is to be maximized.

A conceptually straightforward approach to presenting focus cues is gaze-contingent depth-of-field rendering, where eye-tracking is used to measure where in the scene an observer is fixating, and one of several depth-of-field rendering techniques is used to simulate effects of being defocused with respect to objects at different depths than the fixated point (Mantiuk, Bazyluk, & Tomaszewska, 2011; Duchowski et al., 2014; Maiello, Chessa, Solari, & Bex, 2014; Vinnikov & Allison, 2014). This approach can also be combined with a variable optical element to move the focal distance of the entire display surface to the current fixation distance (so-called varifocal displays) (Wang, Lin, Cakmakci, & Reshetnyak, 2020). This approach addresses the vergence-accommodation conflict and first-order aspects of blur. The second-order aspects of blur are incorrect for everything except the fixated scene point, however, and are instead consistent with the screen surface. For example, in natural viewing outward microfluctuations of accommodation result in increased retinal contrast for farther objects and reduced retinal contrast for nearer objects, whereas here they cause correlated contrast changes across the whole scene independent of depth. A rendering approach called ChromaBlur (Cholewiak et al., 2017) combines manipulation of the on-screen image with a generic model of eye optics to approximate effects of chromatic aberration in the resulting retinal image. Using monocularly viewed scenes, Cholewiak et al. (2017) found this stimulated an accommodation response in the correct direction, and caused images to appear more realistic. Combining ChromaBlur with a varifocal display offers the potential to improve focus cue presentation over “standard” varifocal displays. However, replicating the full suite of second-order blur cues using a gaze-contingent-rendering approach may not be practical. Simulating higher-order aberrations correctly requires knowledge of individual viewer’s eye optics, and simulating the effects of accommodation microfluctuations requires precise real-time measurement of accommodation state.

An alternative approach is fixed-viewpoint volumetric displays, commonly referred to as additive multiple-focal-planes displays (Rolland, Krueger, & Goon, 1999). Here, the eye sees the sum of images displayed on multiple transparent planes positioned at different focal distances (either presented simultaneously using beamsplitter optics (Akeley et al., 2004; MacKenzie et al., 2010), or time-multiplexed, using a variable optical element (Neil, Paige, & Sucharov, 2000; Shevlin, 2005; Liu, Cheng, & Hua, 2008; Love et al., 2009). This approach does not require eye-tracking, and takes advantage of the fact that sensitivity to focal depth is far lower than spatial sensitivity, and so focal-plane resolution can be far lower than pixel resolution at each plane (Akeley et al., 2004). Nonetheless, large numbers of focal planes are impractical, and so such displays typically use three or four focal planes, spaced farther apart than the eye’s depth of focus. Intermediate focal distances are approximated by distributing image intensity across neighboring focal planes at each point in the scene (Akeley et al., 2004; Narain et al., 2015; Mercier et al., 2017). This presents a continuous, and reasonably accurate stimulus to accommodation for focal-plane separations up to ∼0.6 D (MacKenzie et al., 2010; MacKenzie, Dickson, & Watt, 2012). It also produces coarse aspects of defocus blur correctly, in that focusing on a point in the scene ‘naturally’ causes it to become sharper, and focusing at a different distance causes the same point to become blurrier. However, when the eye is focused at intermediate distances both neighboring focal planes are defocused, in different directions, resulting in attenuation of high spatial frequencies (MacKenzie et al., 2010; Ravikumar, Akeley, & Banks, 2011), and non-natural patterns of chromatic aberration and higher-order aberrations. There are also other image artefacts in multiple-focal-planes displays, including incorrect appearance of occlusion edges (Narain et al., 2015).

Light-field (Hainich & Bimber, 2016, ch. 9.5; Lanman & Luebke, 2013) or tensor displays (multi-plane multiplicative displays; Wetzstein et al., 2012; Huang et al., 2015) can reproduce the 4-D light field by emitting different quantities of light in different directions (directional pixels). Achieving high spatial and angular resolution in these displays requires a very large number of individually addressable pixels, however. For this reason, light-field and tensor displays are either designed to provide autostereoscopic viewing (stereo 3-D without glasses and head tracking (Wetzstein et al., 2012), or to provide focus cues (Lanman & Luebke, 2013; Huang et al., 2015), but not both at the same time. The light-field displays that provide focus cues typically need to trade spatial resolution for range of focal distance. Tensor displays can potentially deliver higher spatial resolution, but are limited by diffraction caused by the pixel grid (Huang et al., 2015). In either case, only a coarsely sampled reproduction of the 4-D light field can be generated, making both the first- and second-order blur effects different from those found in the real world.

To determine which of these different display approaches will be effective in presenting highly realistic stereo 3-D imagery, a better understanding is needed of how close an approximation to natural focus cues is required, including which specific aspects of focus cues need to be reproduced and to what degree of fidelity.

### Scene content and the role of focus cues

There may not be a binary answer to the question of whether focus cues are important for perceptual realism, because their contribution is likely to depend on scene content. Our perception of depth is thought to be derived by integrating information from all of the available depth cues, with the weight given to each cue depending on its reliability in a given instance (e.g., Knill & Saunders, 2003; Hillis et al., 2004). This means that, in relatively sparse scenes, with fewer depth cues, the influence of (incorrect) focus cues on perceived depth might be expected to be relatively large compared to in more naturalistic scenes, when the normal complement of depth cues is available (Watt et al., 2005a). It is unclear whether similar principles apply for depth realism. Following the logic of depth-cue integration, it is possible that incorrect focus cues may not have a discernible effect on depth realism when there are many other aspects of scene appearance presented correctly. Alternatively, depth realism could behave differently than expected from cue integration, with unnatural aspects of scene appearance, or motor output, remaining salient even in complex scenes. We examined the effect of focus cues on depth realism in reduced-cue scenes in Experiment 1, and in more realistic scenes in Experiments 2 and 3.

## Experiment 1: Reduced-cue stimuli

The first experiment compared the realism and naturalness of perceived 3-D structure for stimuli presented with fully correct focus cues versus conventional stereo 3-D presentation (holding other aspects of the stimuli constant). We used reduced-cue stimuli—random-dot stereograms—so that any role of focus cues in depth realism could be seen as clearly as possible. Our task was similar to that used by Hibbard et al. (2017), but focus cues were manipulated using a multiple-focal-planes stereoscopic display (MacKenzie et al., 2010; MacKenzie et al., 2012). Our stimuli depicted planar surfaces, and, by moving the focal planes to be precisely coincident with the stimulus planes, we could present focus cues fully correctly (including second-order aspects of blur) rather than merely approximating them. That is, in the correct-focus-cues condition, blur in the retinal image resulted solely from the difference between the distance the observer was focused at and the stimulus distance, including natural effects of their own eye optics. This allowed us to ask the fundamental, proof-of-principle question of whether correct focus cues improve depth realism independent of any implementation-specific shortcomings of focus-cue displays. Conventional stereo conditions were presented using a single focal plane of the display. We expected to find that depth realism i) is increased with correct focus cues compared to with conventional stereo presentation, and ii) does not depend on depth separation.

### Methods

#### Observers

Fifteen observers aged 20 to 32 years (9 females and 6 males) took part in the Experiment. All observers had normal or corrected to normal vision, and normal stereoacuity (assessed by the Randot stereo test; Stereo Optical Company, Inc.). Observers were naïve to the purpose of the experiment, and were rewarded for their participation. Ethical approval for the experiment was given by the School of Psychology and Sport Science Research Ethics Committee at Bangor University.

#### Apparatus

**Figure 1.**
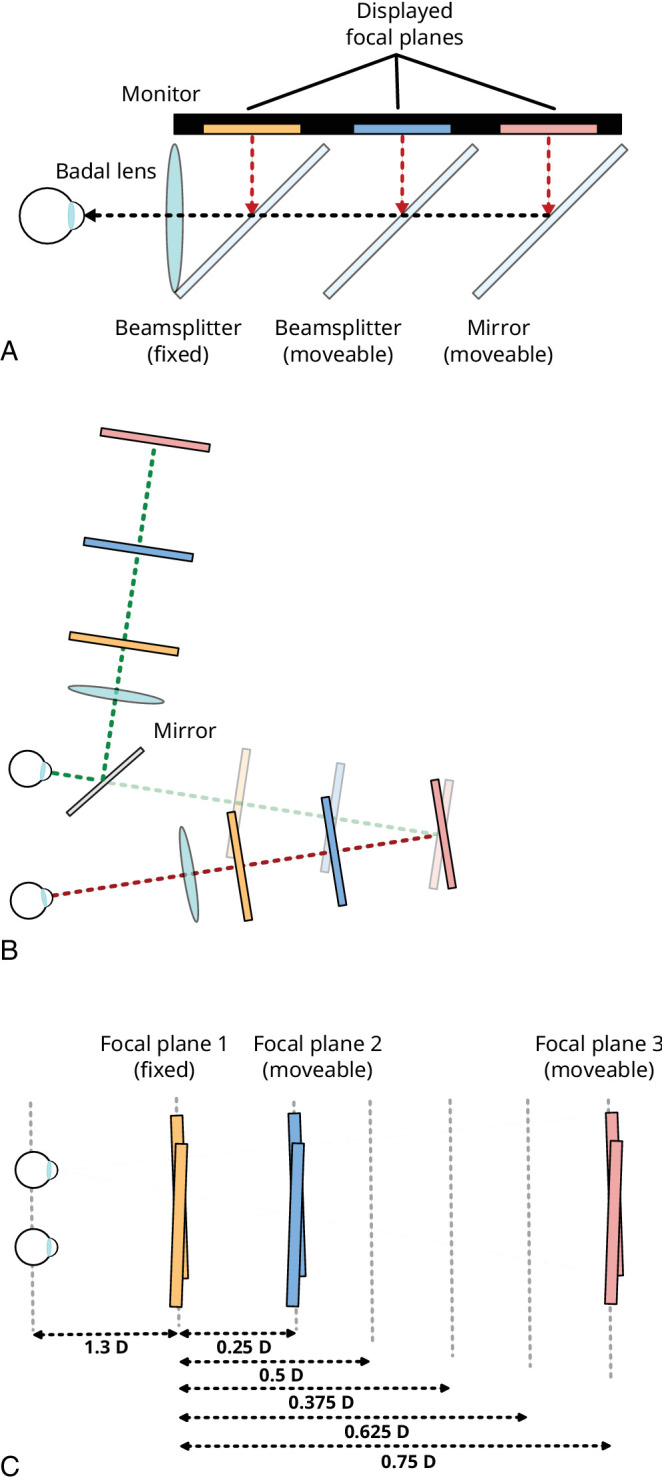
Multiple-focal-planes display for Experiment 1. (A) Side view of the main optical elements. (B) Plan view of the arrangement of left- and right-eye’s displays, and example resulting focal plane positions. The left-eye display is viewed via a mirror, while the right-eye display is viewed directly. The transparent rectangles denote the apparent positions of the left-eye focal planes. The schematic misrepresents the deviation from fronto-parallel, and binocular overlap, of the focal planes: the edges of the stimuli differed from fronto-parallel by just 0.0025 D, and the stimuli had complete binocular overlap. (C) Focal plane positions (and resulting focal-plane separations) used for the correct-focus-cues condition.

The stimuli were presented using the multiple-focal-planes stereoscopic display schematized in Figure 1 (see MacKenzie et al., 2010; MacKenzie et al., 2012, for detailed descriptions). In this display, each eye sees the sum of images presented on three focal planes, created via a combination of two beam splitters and a first-surface mirror. The mirrors and beam splitters are mounted on optical rails, allowing them to be moved to vary the positions of the focal planes. Left- and right-eye images are presented on separate displays, arranged in a haploscope-like configuration. Each display rotates around the eye’s centre of rotation to position the available binocular field of view as needed. The rotation axes were adjusted to be coincident with each observer’s inter-ocular distance (IOD). Images were presented via conventional 30” TFT monitors (Samsung 305T) positioned above the mirrors and beam splitters.

Each eye’s display is configured as a Badal optical system, with images viewed via a spherical lens (1.67 D) placed 60 cm (the focal length of the lens) from the eye (Badal, 1876). Using Badal optics has several benefits for the purposes of this experiment. First, it means that image size, and therefore spatial resolution of the display, is the same at all focal distances (Nyquist limit ∼21 cpd). Second, it allows presentation of large focal-plane separations within a physically small display. Third, focal-plane separations are linear with respect to physical distance, making the precise positioning of focal planes tractable experimentally. Physical constraints of the beam splitter arrangement mean that the nearest focal plane that can be presented is 1.3 D, and the smallest separation between focal planes is 0.25 D. Note, this placed some constraints on which stimuli could be directly compared in the Correct focus cues condition (see *Procedure*). The observer’s head was stabilised and positioned with respect to the apparatus using a viewport attached to the apparatus that resembled a rigid pair of goggles, with separate left- and right-eye circular viewing apertures. This viewport fitted snugly around the observers’ eyes/face when leaned against, maintaining a stable position. Distance to the Badal optics was controlled by aligning the outer (lateral) canthus—the point where upper and lower eyelids meet, which corresponds approximately to the eye’s nodal point (Elliott, 2007)—with a reference marker on the apparatus.

Luminance and color calibration were performed such that stimulus elements had the same luminance (15 cd/m^2^, background 0.27 cd/m^2^) and were “white” (i.e., spectrally broadband; CIE 1931 2° chromaticity coordinates, *x* ≈ *y* ≈ 0.333 (see MacKenzie et al., 2010, for details) on all focal planes in both eyes’ displays. Calibration was performed using a Minolta CS-100 Chromameter, with measurements taken at the eye’s position, viewing through all the optical elements of the display. The focal planes that moved during the study (Figure 1C) were separately calibrated at each of their possible positions to account for any spatial non-uniformities in monitor output. Images were presented using MATLAB (Mathworks) and Psychtoolbox (version 3.0.8; Brainard, 1997).

This display affords independent manipulation of distance specified by disparity and by focus cues while holding all other stimulus aspects constant. Conventional 3-D presentation is achieved by presenting images on a single focal plane and correct focus cues are achieved by presenting stimuli on focal planes at different distances, positioned as appropriate.

#### Stimuli

**Figure 2.**
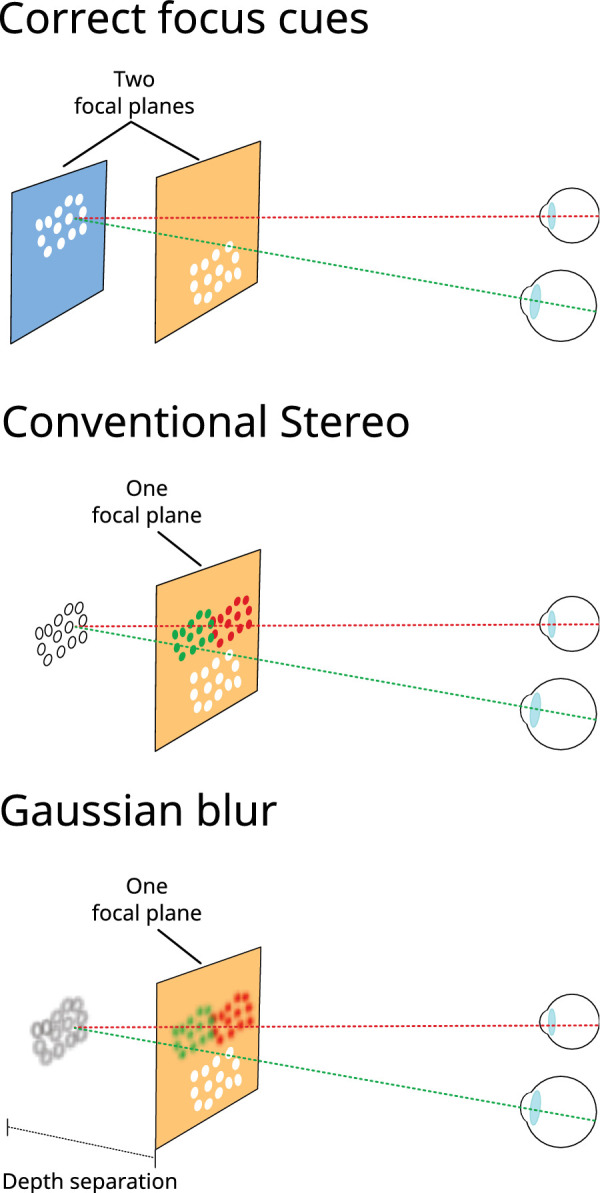
Focus-cue conditions in Experiment 1. In the Correct focus cues condition (top) near and far random-dot rectangles were presented on separate, correctly positioned focal planes (in this example, the near and middle focal planes, but some stimuli were presented on near and far planes). In the other two conditions (middle and bottom) both the near and far random-dot rectangles were presented on a single focal plane (the near one). Green and red colors denote the dots in the left- and right-eye images, respectively (the dots in the actual stimuli were white).

On each trial observers viewed two stimulus intervals, with each interval comprising a pair of random-dot-defined fronto-parallel rectangles, vertically separated, and with varying depth separations between them specified by binocular disparity (Figure 2). Stimulus size was limited by the field of view of the display, which is constrained by the physical size of the Badal lenses. The individual stimulus rectangles were on average 1.6° high and 6.0° wide. A random jitter was added in the range ±0.25° for the height dimension, and ±1.5° for width (drawn from a uniform distribution) to prevent relative size of the rectangles providing a reliable cue to depth separation. The vertical separation of the two rectangles was on average 0.3°. The random dots were circular (diameter = 11.5 arcmin), and the dot density was 3.0 dots per degree^2^. To avoid clustering of dots, dot positions were not fully randomised but were instead determined by jittering a regular grid. Random (uniform distribution) vertical and horizontal shifts were added to each dot in the range ±0.3 times the initial grid spacing (∼0.58°). Dots were rendered with anti-aliasing. Whether the upper or lower rectangle was nearer was chosen at random on each stimulus interval. We used vertically separated stimuli rather than overlapping ‘transparent’ stimuli (as used by Hibbard et al., 2017) to avoid occlusions, which multiple-focal-planes displays do not present correctly Narain et al., 2015). Disparities were calculated taking individual observers’ IODs into account.

In each stimulus interval the two rectangles were presented in one of three focus-cue conditions, described below (and shown in cartoon form in Figure 2).

• Correct focus cues. Both nearer and farther random-dot rectangles were presented at focal distances that precisely coincided with their disparity-specified distances. Focus cues were, therefore, consistent with viewing an equivalent stimulus in the real world.
• Conventional stereo. Both nearer and farther rectangles were presented on a single display surface—the near focal plane in our display—positioned at 1.3 D (∼77 cm). This condition, therefore, replicated key aspects of conventional stereo 3-D presentation.
• Gaussian blur. Here, both rectangles were again presented on the near focal plane, but Gaussian blur was applied to the dots on the far rectangle. This blur depended on depth separation, but did not change when the far rectangle was fixated, and so was highly unrealistic in appearance. This condition was intended as a foil to detect observers preferentially choosing as more realistic stimuli that contained *any* visible blur, independent of depth realism (see below).

Note that in all three focus-cue conditions the near rectangle was always at the same disparity-specified and focal distance, coincident with the near focal plane (which was fixed at 1.3 D [∼77 cm]; Figure 1C). Variations in depth separation between rectangles were achieved by moving the far rectangle, which meant that only the far rectangle was presented differently across different focus-cue conditions.

We were concerned that a false-positive result for correct focus cues (vs. conventional stereo) could arise from observers simply responding preferentially to the presence of discernible blur in the stimulus, irrespective of depth realism. This was a particular concern because random-dot stereograms are themselves unrealistic, which could make it more difficult for observers to maintain an appropriate internal standard for judging depth realism. The rendered Gaussian blur condition was a foil, designed to detect such a pattern of responses by presenting focus cues in an exaggeratedly unrealistic way. For each depth separation, we applied Gaussian blur to the far rectangle creating an approximate empirical match to the real blur caused with correct focus cues when fixating the near rectangle. This resulted in obviously incorrect blur when observers moved fixation between near and far stimuli (see *Procedure*) because, regardless of fixation, the near rectangle remained sharp and the far rectangle remained blurred. We reasoned that, if observers systematically chose obviously unrealistic blur as having higher depth realism, we could not trust that their judgments of correct-focus-cues stimuli reflected depth realism per se, and so they should be removed from the analysis (see *Results*). Although the rendered Gaussian blur condition does not speak to our hypotheses, we also included it in the magnitude-judgment variant of the experiment so that any difference in patterns of effects of correct focus vs. conventional stereo across magnitude and depth-realism judgments could not be due to the inclusion of different conditions.

#### Procedure

Observers completed two variants of the experiment: one in which they judged depth magnitude, and one in which they judged depth realism. All other aspects of the two variants were identical. Observers fully completed one variant before undertaking the other. Eight observers completed the depth-magnitude variant first and seven completed the depth realism variant first.

At the beginning of each experiment block observers checked and if necessary adjusted their head position vertically and horizontally to ensure their eyes were correctly centered on each eye’s display. To do this, dots were presented to both eyes on the geometric centres of each focal plane and observers adjusted their position until the three dots in each eye were superimposed. The experimenter also checked that the observer’s eyes were the correct distance from the Badal optics (see *Apparatus*).

Each trial consisted of two stimulus intervals, each containing a pair of random-dot rectangles separated in depth (see *Stimuli*). The pairs of stimuli could be drawn from the same or different focus-cue conditions. On each trial observers made a two-interval, forced-choice response (via a gamepad). In the depth-magnitude variant they indicated which interval contained the largest depth separation between the pairs of rectangles. In the depth-realism variant, observers indicated which interval contained the most realistic depth separation. Depth realism was explained to the observers as relating to how tangible, solid and real they felt the separation in depth was. This terminology was based on the depth-realism task used by Hibbard et al. (2017), and is derived from an analysis of the phenomenology of 3-D perception (Vishwanath & Hibbard, 2013). Each stimulus interval was presented for 3 seconds with a 1-second inter-stimulus interval (blank screen). Observers were instructed to look around the scene and fixate both stimulus rectangles sequentially, to ensure dynamic patterns of accommodation response and blur were generated, as in real-world viewing.

Depth separations of 0.25, 0.375, 0.5, 0.625, and 0.75 D were presented in all three focus-cue conditions. Depth separations were defined relative to the fixed, near focal plane at 1.3 D (Figure 1C). They correspond to disparities of ∼27, 53, 80, 106, 133, and 160 arcmin (assuming a 62-mm IOD). All possible pairings of depth separation and focus-cue condition were presented, within and across focus-cue conditions, with the exception of “adjacent” pairs of depth separations in the correct-focus-cues conditions (0.375 D vs. 0.5 D, for example), which could not be presented due to the minimum focal-plane spacing of 0.25 D (see *Apparatus*). This resulted in a total of 101 pairwise comparisons. The mid and far focal planes were manually moved to present different depth separations in the correct-focus-cues conditions. A given positioning of the focal planes allowed two depth separations per experiment block. This meant that stimulus presentation could not be fully randomized. Instead, each block contained two correct-focus-cues depth separations, and the remaining trials (within and across conventional stereo and rendered Gaussian blur conditions) were randomly distributed across blocks. Overall, observers completed 20 repetitions of each stimulus pair, divided across 6 blocks of trials, making 2,020 trials in total for both depth-magnitude and depth-realism variants of the experiment. Each observer completed the experiment across multiple days.

### Results

We derived Thurstone “scale values” (Thurstone, 1927) for each observer, for both their depth magnitude and depth realism judgments. For each pair-wise comparison, we calculated the proportion of times they chose a given stimulus interval (collapsed across whether the far rectangle was at the top or bottom). We then used Bayesian maximum likelihood estimation under Thurstone’s case V conditions to calculate each observer’s scale values for each depth separation in each focus-cue condition (Tsukida & Gupta, 2011; Perez-Ortiz & Mantiuk, 2017) using *pwcmp* software.^1^

**Figure 3.**
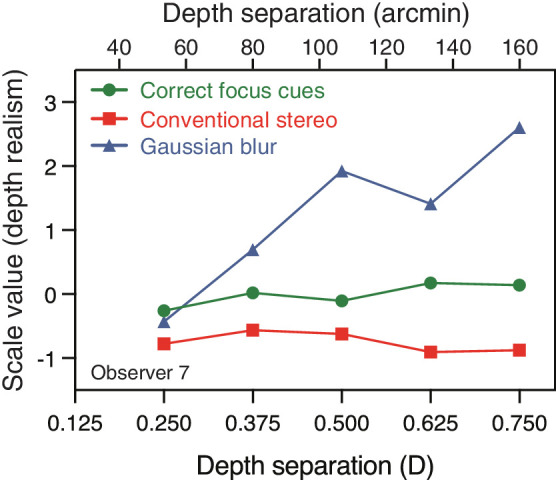
Depth realism judgments for an example observer who clearly selected rendered Gaussian blur stimuli as having more depth realism than the other conditions. The figure plots this observer’s Thurstonian scale values for depth realism judgments as a function of depth separation for each focus-cue condition. Higher values indicate higher perceived realism. The upper x-axis shows depth separation in terms of disparity (arcmin). These values are inexact because the disparity projected by a given depth separation (at a given distance) depends on observer IOD whereas depth separation in diopters does not. We assumed an IOD of 62 mm when making the conversion.

We first examined each observer’s data for evidence that they had responded preferentially to the rendered Gaussian blur foil condition when making realism judgments. Three observers clearly showed this pattern, systematically judging the foil condition as having higher depth realism than the conventional stereo condition. Figure 3 shows an example observer who showed this pattern (see Appendix for individual data). Based on the logic outlined previously, we deemed these observers likely to have selected stimuli containing discernible blur regardless of its effect on depth realism (note, two of the three also selected correct focus cues over conventional stereo) and so they were removed from subsequent analyses. All three also showed a systematic increase in likelihood of selecting the foil condition as the amount of blur increased (with increasing depth separation). This is consistent with the amount of blur being used as a simple proxy for depth realism, because the stimulus appearance was arguably increasingly unrealistic as blur increased (when the far rectangle was fixated). Note, excluding these three observers slightly affected our control for order effects: in the final data set seven observers completed the magnitude judgement first and five completed the realism judgment first.

**Figure 4.**
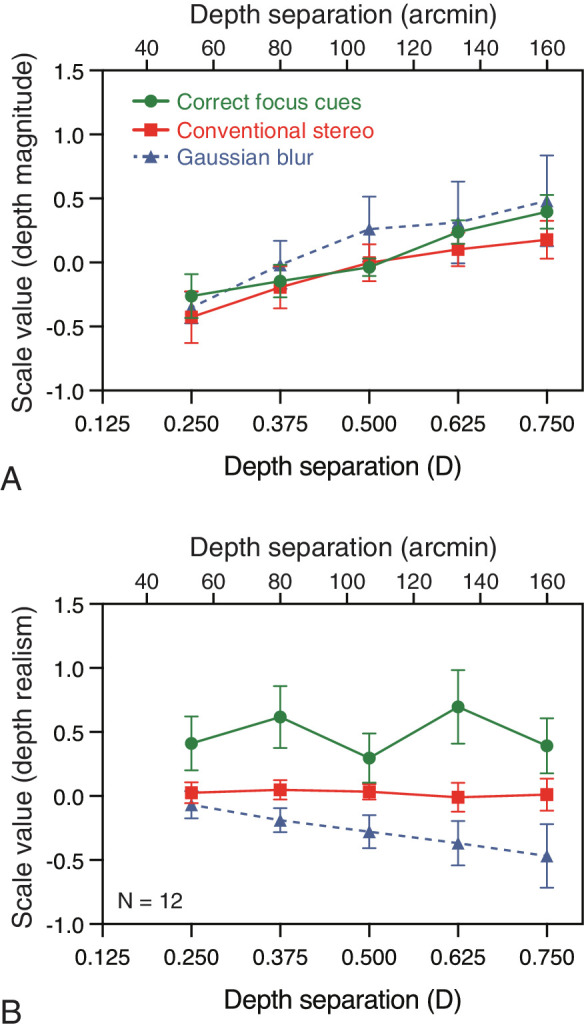
Experiment 1 results. Average scale values for (A) depth magnitude and (B) depth realism judgments as a function of depth separation, for the three focus-cue conditions. The upper x-axis shows depth separation in terms of disparity, calculated as per Figure 3. Error bars denote ±1 SEM.

We averaged across the remaining 12 observers to give mean scale values for depth magnitude and depth realism judgments. Figure 4 shows the results for both types of judgments as a function of depth separation between stimulus rectangles. Our analysis concentrates on the results of correct-focus-cues vs. conventional stereo presentation (Figure 4 also shows the results in the Gaussian blur foil conditions for inspection purposes).

Perceived depth magnitude (Figure 4A) increased systematically as a function of increasing depth separation in both the conventional stereo and correct-focus-cues conditions. We did not find a roll-off in perceived depth at larger depth separations (c.f. Hibbard et al., 2017). There was no evidence of an effect of focus-cues condition on depth magnitude. Consistent with this, a two-way (depth separation ×focus-cue condition) repeated measures analysis of variance found a significant main effect of depth separation on perceived depth magnitude, *F*(4, 44) = 7.92, *p* < 0.001, but no significant main effect of correct-focus-cues vs. conventional stereo presentation, *F*(1, 11) = 0.49, *p* = 0.50, and no significant interaction, *F*(4, 44) = 1.52; *p* = 0.21. These results indicate that while perceived depth increased reliably with disparity-specified depth separation, the magnitude of perceived depth was unaffected by focus-cue condition.

For depth realism judgments, correct focus cues were systematically judged as more realistic than conventional stereo presentation (4B). This is consistent with correct focus cues providing a benefit to perceptual realism. Depth realism was unaffected by depth separation, however. This was the case not only in the correct-focus-cues condition (as predicted), but also in the conventional-stereo condition (i.e., we did not replicate the roll-off in depth realism reported by Hibbard et al., 2017, with conventional stereo presentation). A two-way (depth separation × focus-cues condition) analysis of variance confirmed there was a significant main effect of correct focus cues vs. conventional stereo, *F*(1, 11) = 5.08, *p* < 0.05, no significant main effect of depth separation, *F*(4, 44) = 0.78; *p* = 0.55, and no significant focus cues × depth separation interaction, *F*(4, 44) = 1.16; *p* = 0.34. These results show that correct focus cues did result in increased perceived realism of depth separations compared to conventional stereo 3-D presentation. We discuss the consistency of this effect across individual observers in the *Discussion*.

### Discussion

#### Summary of results

Experiment 1 addressed the question of whether perceived 3-D structure is more realistic when focus cues are correct compared to when using conventional stereo presentation (with incorrect focus cues), using reduced-cue stimuli to isolate their contribution. We found that this was the case, providing proof-of-principle evidence that correct focus cues can contribute to increased depth realism. We found no effect of correct focus cues on perceived magnitude of depth separation. The markedly different pattern of effects of correct vs. incorrect focus cues across depth magnitude and depth realism judgments suggests that observers were able to respond selectively to different dimensions of their perceptual experience in the two tasks (Hibbard et al., 2017). Taken together, these findings suggest that correct focus cues may be necessary to maximise realistic, natural appearance of depth in stereo 3-D. We explore this further in Experiments 2 and 3.

#### Relationship between focus cues, depth separation, and depth realism

Depth realism did not decrease with increasing depth separation when focus cues were presented correctly (c.f. Hibbard et al., 2017), but instead remained on average constant. As described in the Introduction, this is in keeping with the intuition that the realism of real-world scenes would not normally be affected by depth and distance relations. We cannot, however, conclude unambiguously that the roll-off in depth realism with depth separation reported by Hibbard et al. (2017) was an artefact of using conventional stereo presentation because depth separation also did not affect depth realism in our conventional stereo condition (i.e., we did not replicate the roll-off found by Hibbard et al. (2017). One reason for this difference may be that Hibbard et al. (2017) used overlapping, or transparent stereoscopic stimuli. Stereo transparency presents a greater challenge for stereoscopic fusion than our non-overlapping stimuli due to the prevalence of false matches in the two eye’s images (Akerstrom & Todd, 1988; Tsirlin, Allison, & Wilcox, 2008), which could cause stereoscopic fusion to break down at smaller depth separations than in our study. Moreover, although we routinely experience unfused binocular images in natural viewing, failure to fuse transparent stereo stimuli arguably results in a particularly confusing appearance, that may be uncommonly experienced in real-world viewing, causing it to be judged less realistic. Thus, while our experiment does show that correct focus cues increase depth realism over conventional stereo presentation, we cannot disambiguate whether the roll-off in depth realism with increasing depth separation observed by Hibbard et al. (2017) for their transparent stimuli was caused by incorrect focus cues (as we hypothesised) or by the overlapping nature of their stimulus.

In our experiment, correct focus cues conferred a similar increase in depth realism independent of depth separation. This finding is perhaps surprising given that the error in focus cues in conventional stereo presentation is larger with increasing depth separation. It is possible, however, that any detectable error in focus cues reveals that they are non-natural, resulting in a similar reduction in realism independent of the magnitude of the error. Even at the smallest depth separation we tested (0.25 D), it is likely that the blur in the non-fixated stimulus rectangle was detectable, for example, given the eye’s effective depth of focus with a large pupil size (Campbell, 1957; Charman & Whitefoot, 1977; Green et al., 1980). Moreover, as discussed in the Introduction, second-order aspects of blur, and motoric responses from accommodation, may also have contributed to discriminating between correct and incorrect focus-cue conditions. It is also possible (at least for larger depth separations) that the far stimulus rectangle in the Conventional stereo condition could have appeared unnaturally blurred when fixated, owing to inaccurate accommodation caused by vergence-accommodation conflict. Because our experiment compared fully correct focus cues to conventional stereo presentation we cannot determine the contribution of each of these signals to depth realism.

#### Individual differences

Although there was a statistically significant increase in depth realism with correct focus cues, individual observer’s data show individual differences in the consistency of those effects (see Appendix). Of the 12 observers entered into the analysis, two showed large and highly consistent increases in depth realism with correct focus cues, while a further four showed consistent increases, but smaller in magnitude. Four observers showed large effects at a single depth separation, and a further one observer showed no consistent pattern of effects. Finally, just one observer showed a consistent *decrease* in depth realism with correct focus cues. Taken together, these data suggest that the degree to which presenting correct focus cues improves depth realism is not consistent across different individuals, indicating that the weight given to focus cues in this aspect of perceptual realism is quite variable.

#### Depth magnitude judgments

Presenting correct focus cues had no effect on perceived depth magnitude. This finding is largely consistent with previous studies examining direct effects of focus cues on properties, such as perception of disparity specified slant (Watt et al., 2005a; Watt et al., 2005b), although note there is evidence that focus cues can affect perceived depth from disparity indirectly, via the distance estimate used to interpret binocular disparities (Watt et al., 2005a; Hoffman et al., 2008).

Perceived depth magnitude increased linearly with depicted depth separation in both the correct-focus-cues and conventional stereo conditions, throughout the range of separations tested, rather than rolling-off at higher depth separations (depth magnitude rolled-off around 0.4 D in Hibbard et al., 2017). Hibbard et al. (2017) concluded that in their case this was due to the stimuli exceeding binocular fusional limits, which would also be exceeded in our experiment. Our lack of roll-off in depth magnitude with correct focus cues could, in principle, be attributed to stereoscopic fusion being easier when focus cues are presented correctly (Hoffman & Banks, 2010). This cannot explain why we found a similar result in our conventional stereo condition, however, suggesting other differences between the two studies caused the different patterns of findings. For instance, as noted, our stimulus rectangles were non-overlapping, rather than transparent, and stereo fusion might be expected to break down sooner in the latter case (Akerstrom & Todd, 1988; Tsirlin et al., 2008). Also, our observers were explicitly instructed to look between the two rectangles, making sequential vergence eye movements, which could have provided information about relative depth magnitude across stimulus intervals even if the non-fixated stimulus could not be fused (Enright, 1991).

## Experiment 2: Realistic stimuli

**Figure 5.**
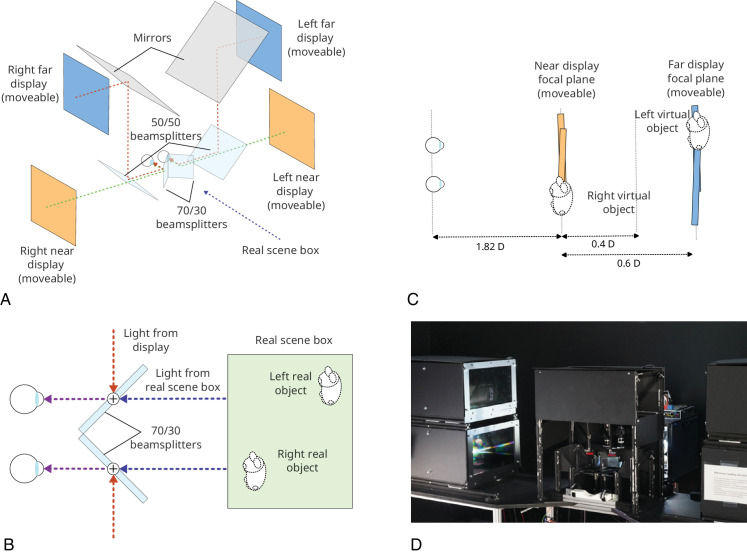
Display used in Experiments 2 and 3 (diagrams not to scale). (A) The physical configuration of mirrors, beam splitters and HDR displays. (B) The configuration of the real-scene box in relation to the display. (C) Unfolded views showing the possible focal plane positions used in the experiment. (D) Photograph of the display.

We next examined whether correct focus cues increase depth realism in more complex, realistic imagery, containing an almost complete set of visual cues. To do this we used a high-dynamic-range stereoscopic display with two focal planes, that was developed specifically to present highly realistic imagery (Zhong et al., 2021; see Figure 5). We examined the effect of focus cues at low and high luminance levels, which is practically useful because the smaller pupil size at high luminance might be expected to further reduce any effects of focus cues by increasing the eye’s depth of focus. We also examined two depth separations because even though varying depth separation had no effect on realism in Experiment 1, we wanted to check that this observation also holds for more complex stimuli. We presented two objects, one coincident with the position of each focal plane (similar to Experiment 1). As well as comparing correct focus cues with conventional stereo presentation, we also examined the efficacy of two approaches to simulating blur via rendering: a simple first-order (i.e., defocus) model of retinal image blur, and ChromaBlur (Cholewiak et al., 2017). In both cases, we used a simple gaze-contingent depth-of-field rendering approach, in which rendered blur was applied to the non-fixated object, but the physical focal distance to all image points remained constant (i.e., images were presented on a single, fixed display plane). The efficacy of the varifocal approach, which combines gaze-dependent rendering with changing the focal distance of the display to match fixation distance, is examined in Experiment 3. A final goal of Experiment 2 was to better understand the magnitude, and therefore likely importance, of any effects of focus cue presentation on depth realism by comparing them to effects of reducing other, more familiar aspects of image quality. To do this, we examined the effect of reducing spatial resolution, reducing contrast, and of 2-D binocular presentation. We did not measure perceived depth magnitude in Experiments 2 and 3.

### Methods

#### Observers

Fourteen observers aged 18 to 26 years (5 males, 9 females) took part in the experiment. All observers had normal or corrected to normal vision, normal stereoacuity (assessed by the Titmus stereo test) and normal color vision (assessed by the Ishihara test). Observers were naïve to the purpose of the experiment, and were rewarded for their participation. The experiment was approved by the Ethics Committee of the Department of Computer Science and Technology, University of Cambridge.

#### Apparatus

The high-dynamic-range, multiple-focal-planes stereoscopic display is schematized in Figure 5 (see Zhong et al., 2021 for full details). Each eye’s display consists of two focal planes, each produced by a separate 9.7” HDR display. The individual HDR displays comprise a 2,048×1,536 px LCD (LP097QX1) with the backlight removed, and substituted with a DLP projector (Acer P1276; 1,024×768 px) projecting the light on a diffuser and a Fresnel lens behind the LCD panel. By separately modulating the light emitted by the projector and the transparency of the LCD (as explained in Seetzen et al., 2004), we could achieve both very high contrast (more than 1,000,000:1) and high peak luminance (over 4,000 cd/m^2^) and almost negligible black level (<0.01 cd/m^2^). To improve the light efficiency of the system, we removed the color wheel from the projectors.

Within each eye’s display, 1-mm plate beam splitters and first-surface mirrors were used to combine images from two HDR displays to present images at two focal depths. The left- and right-eye displays were viewed via beam splitters in front of the eyes, via a conventional Wheatstone-stereoscope configuration. The display did not include any lenses or waveguides, which could introduce aberrations or other visual artefacts in the displayed image. As well as providing variations in focal distance, this display has high spatial resolution (Nyquist limit ∼50 cpd at the near plane). The display was calibrated to reproduce linear RGB values in the BT.709 color space and all rendering, including focus cue simulation, was performed in that space. Color was measured (with a JETI Specbos 1211) through the entire optical stack to compensate for inconsistencies in spectral reflectance.

The display also has a real-scene box (Figure 5B) that allows real, physical objects to be viewed in the same 3-D space as the virtual objects, through the beam splitters in front of the eyes (see *Procedure*). Visibility of real objects is controlled by switching on and off lights in the roof of the real-scene box.

#### Stimuli

**Figure 6.**
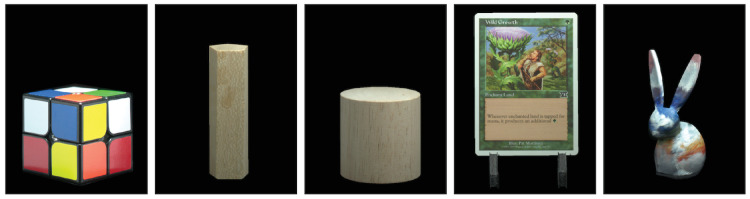
Renderings of the objects used to construct scenes displayed during Experiment 2.

On each trial, we showed two objects, presented side by side, with the object on the right closest to the observer, as shown in Figure 5C. The stimuli were highly realistic renderings of real objects. The objects used in Experiment 2 are shown in Figure 6. An example stimulus is shown in Figure 8. The bases of the objects were positioned at 50 mm below eye level and they were separated horizontally by 6.6° of visual angle.

**Figure 7.**
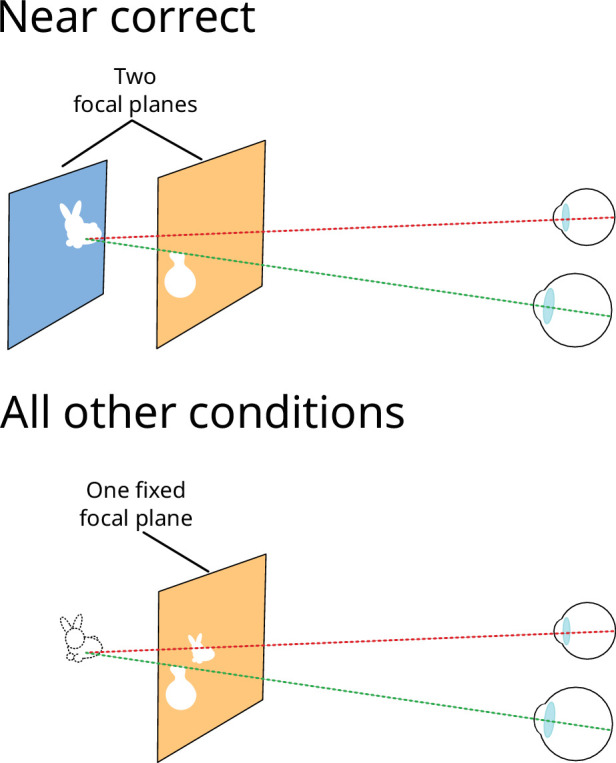
Use of focal planes in Experiment 2. The Near correct condition used both focal planes of the display and all other conditions used only the near focal plane.

**Figure 8.**
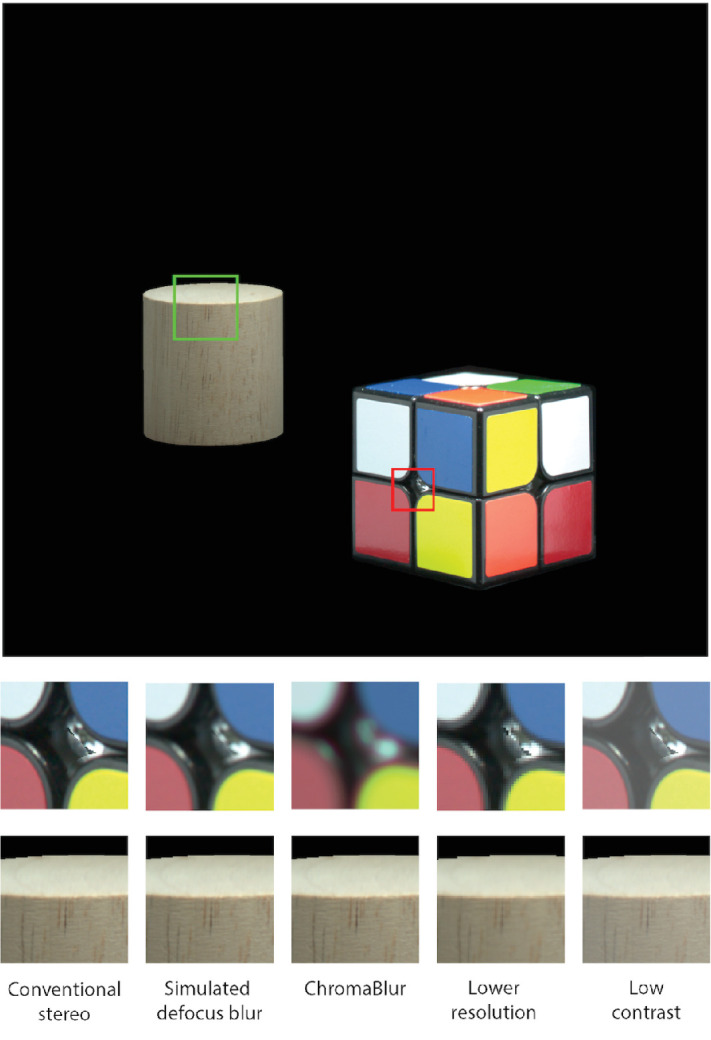
Example of rendered output for Experiment 2. Main image: near (right) and far (left) objects, rendered in-focus. Small images: close-ups of near and far objects in the various rendered-blur and reduced-quality conditions, assuming the far (left) object is fixated. In the Zero disparity condition (not shown) both objects were rendered in-focus as per Conventional stereo. In the Near-correct condition (not shown) both objects were also rendered in-focus, but displayed on different focal planes so that natural accommodation caused appropriate blur.

The procedure used to capture and render the images is described in detail in Zhong et al. (2021). Briefly, HDR images of the objects were captured from multiple viewpoints and rendered using the Lumigraph rendering technique (Gortler, Grzeszczuk, Szeliski, & Cohen, 1996). The technique involves projecting the captured HDR images on the surface of the object’s 3-D model. To correctly reproduce viewpoint-dependent changes in 3-D object appearance (e.g., glossy reflections), we projected the image that was captured closest to the viewing axis of each eye (determined in the calibration procedure, as explained below). The Lumigraph rendering technique lets us interpolate between the captured views, adjust rendering for each observer’s IOD, and render accurate 3-D geometry as seen from the viewpoint of each observer.

Stimuli were presented in the following focus-cue conditions.

• Near-correct focus cues. The near object was presented on the near focal plane and the far object on the far focal plane (see the upper portion of Figure 7). Note, focus cues in this condition were *near* correct because, unlike in Experiment 1, the objects had depth relief, and so their surfaces were not precisely coincident with their respective focal planes. This discrepancy was small, however (typically 0.15 D at the near plane), and the overall difference in focal distance to the two objects was accurately reproduced.
• Conventional stereo. Both objects were presented on the near focal plane, reproducing correct geometrical cues, but with incorrect focus cues for the far object (bottom of Figure 7). Because it reproduces standard stereo 3-D presentation this condition is a baseline against which to compare the other conditions.
• Simulated defocus blur. As in Conventional stereo, both objects were presented on a single plane but depth-dependent defocus blur was applied (as described elsewhere in this article), assuming that the currently fixated object was correctly focused (see *Eye tracking*).
• ChromaBlur. As Simulated defocus blur but rendered blur was added using the ChromaBlur algorithm (Cholewiak et al., 2017), which simulates both defocus blur and chromatic aberration.

We also presented stimuli in three degraded-quality conditions:

• Lower-resolution. As Conventional Stereo, but content was rendered at half resolution (50 ppd; Nyquist frequency of 25 cpd), and up-scaled to the display resolution using nearest-neighbor filtering (to simulate larger pixels).
• Low-contrast. As Conventional Stereo, but altered to simulate the appearance of a display with a lower dynamic range. This was achieved by adding 10% of the mean luminance value to the linear color value of each pixel, which results in an elevated black level and reduced contrast in dark areas.
• Zero disparity. Images were presented only on the near focal plane and the objects were rendered from a single viewpoint, the Cyclopean eye (the mid-point of the observer’s measured eye positions). This condition therefore reproduced a binocularly viewed conventional 2-D display. It served primarily as a rationality check because depth realism should be greatly reduced with no disparity-specified depth separation between objects.

The angular spatial resolution of the far focal plane, used in the Near-correct focus cues condition, was higher than for the near focal plane because it was farther away. This increased resolution, in and of itself, could lead to higher depth realism ratings in the Near-correct focus cues condition (i.e., a false positive). To control for this factor, we downsampled the images presented on the far focal plane using a box filter such that the effective angular resolution of the stimuli was matched across focal planes and conditions.

In the Simulated defocus blur and ChromaBlur conditions, we simulated defocus blur by performing depth dependent filtering of the image generated by our rendering pipeline. We filtered each pixel by a blur kernel that depended on the condition type. We used a cylinder function with diameter, *K*_d_, in angles, calculated as:

(1) $$\begin{array}{c} K_{d}=\frac{180}{\pi }\;10^{-3}\;P\;\left|D_{f}-D_{p}\right|,\; \end{array}$$

where *P* is the diameter of the observer’s pupil in millimeters, *D*_f_ is the depth of the focal point in diopters and *D*_p_ is the depth of the pixel in diopters (Strasburger, Bach, & Heinrich, 2018) (note, we converted from radians to degrees, and from millimeters to meters, using the constants $\frac{180}{\pi }$ and 10^−3^, respectively). The pupil diameter was estimated for each observer as a function of their age and estimated luminance of the stimuli (between 5.2 and 5.4 mm at 100 cd/m^2^ and 7.2 and 7.5 mm at 1 cd/m^2^), using the formula derived in Watson and Yellott (2012). We also validated those values by measuring the pupil (on a photograph with a millimetre scale) of three observers while they were observing the stimulus. When filtering background (black) pixels, we set *D*_*p*_ to be either the depth of the near or the far focal plane, determined by which object the pixel was closer to. This allowed us to approximate the blurred fringes of objects.

Additionally, in the ChromaBlur condition, we simulated chromatic aberration using the technique outlined in Cholewiak et al. (2017). In our implementation, we performed defocus filtering on each color channel individually and shifted the depth of the filtered pixel according to the difference between displayed and in-focus wavelengths of light. As in the original method, we approximated chromatic aberrations by superimposing the simulation for three color channels, using wavelengths corresponding to the peaks of the corresponding spectral emissions of our display (measured with a spectroradiometer).

An important distinction of the ChromaBlur condition is that it compensates for natural aberrations introduced by the viewer’s eye when viewing the image on our display. The goal is not to display retinal images (as the Simulated defocus blur does), but to display images that would result in a correct image on the retina when passed through the eye’s optics. As in Cholewiak et al. (2017), this is achieved by a deconvolution pass on the target retinal image. Because such deconvolution is computationally expensive, we generated the required images offline after calibration but prior to the experimental trials, based on the determined pupil diameter. Our implementation used Wiener deconvolution, calculating the per-channel blur kernel as in Cholewiak et al. (2017), with the focal point set to the near focal plane of our display, and assuming additive noise with a mean of 0 and a variance of 12 · 10^−8^.

In conditions with simulated focus cues, when fixation shifted from one object to another we did not present an abrupt change in blur. Instead, we linearly interpolated between the previous and current focus distance over a period of 200 ms, mimicking smooth transitions caused by natural accommodation (Heron, Charman, & Schor, 2001).

#### Eye-tracking

We used a custom-built eye-tracker to estimate gaze position in the two rendered blur conditions: Simulated defocus blur and ChromaBlur. Commercially available eye-trackers could not easily be fitted within the confined space of the display, and precision requirements were relatively low because we only needed to identify which of two separated objects was fixated at a given moment. The eye tracker consisted of an IDS UI-3140CP-M-HQ Rev.2 monochrome computer vision camera (sensitive in the infrared band) monitoring the eye via a hot mirror. Six infrared LEDs illuminated the eye and provided corneal reflections, which could be tracked together with the pupil. The eye-tracker was calibrated for each observer as follows. First, we presented 16 crosses at pre-defined positions in a random sequence, one after the other, for a period of 3 seconds each. We then fitted a multivariate polynomial that mapped pupil and corneal reflections to the gaze direction.

#### Observer alignment

**Figure 9.**
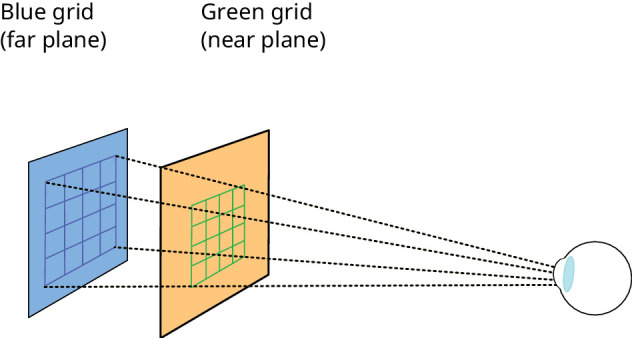
Cartoon of observer alignment procedure. For each eye, the grid on the near focal plane was aligned with that on the far focal plane.

For the images to be correct, the observers’ eye position needed to be known, and controlled. The head was stabilised using a combination chin and forehead rest, and eye position was determined using a short calibration procedure conducted before each session. Separately with each eye, observers were asked to align green and blue grids, shown on the near and far focal planes, respectively (see Figure 9), by using a computer mouse to drag the corners of the near grid to align with the far grid. Because the physical properties of the display are known precisely, the resulting settings could then be used to determine the position of each eye, and observer IOD.

#### Procedure

At the start of each testing session, observers completed the alignment and eye-tracking calibration procedures. Observers then performed pairwise comparisons between pairs of conditions selected using an adaptive procedure (active sampling, explained below). The different depth separations and luminance levels were tested in separate blocks. Observers saw one condition from a pair at a time and could switch between them using keys. The compared pairs of conditions contained the same near and far objects, but objects for each comparison were selected randomly. The observers were asked to select the condition from a pair that contained the most realistic depth. They confirmed their choice by pressing another key while their selected condition was displayed. Similar to Hibbard et al. (2017), presentation time was not limited. As in Experiment 1, observers were instructed to base their judgments on how tangible, solid and real the depth appeared (Vishwanath & Hibbard, 2013).

**Figure 10.**
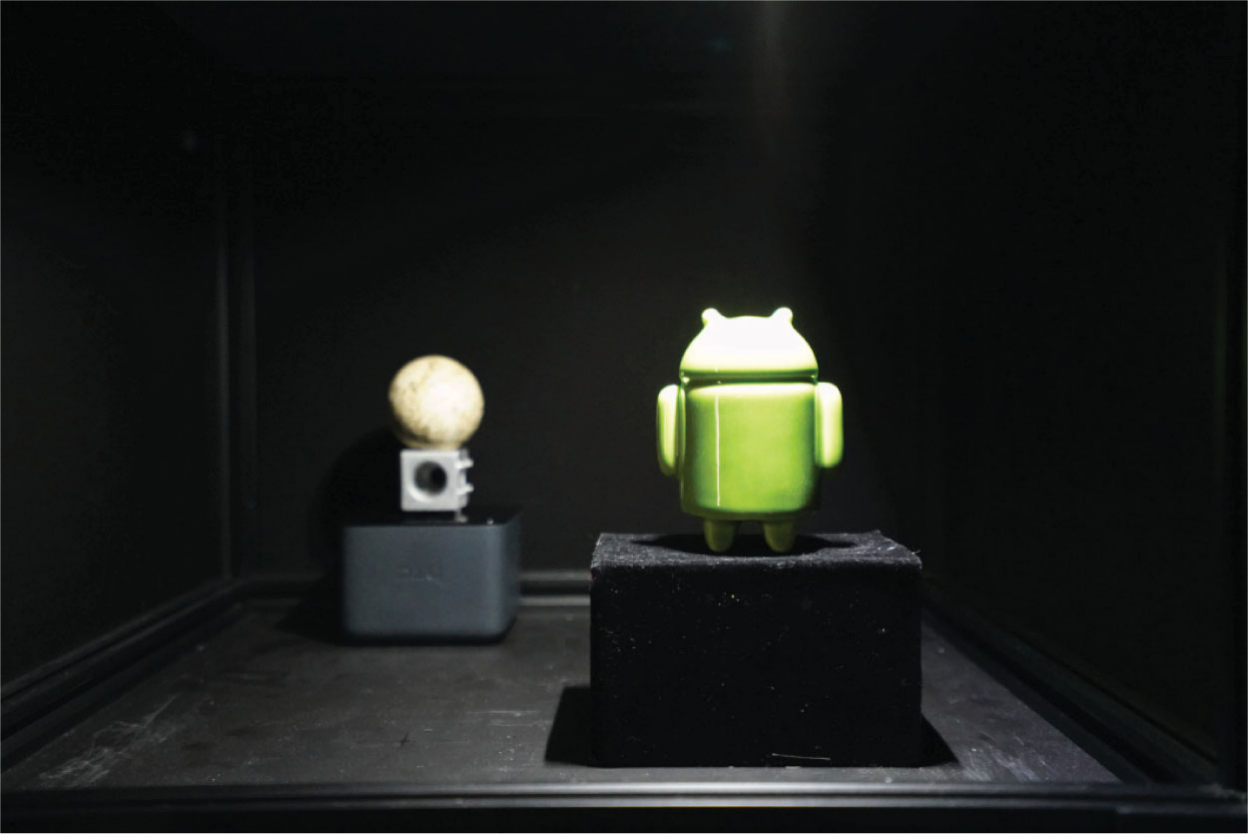
Photograph of the real scene, used to calibrate observers’ understanding of the term “depth realism.”

To help observers understand what was meant by “depth realism,” and to help them maintain a calibrated internal standard, at the beginning of each session, and after every 10 pair-wise comparisons thereafter, observers saw a real, physical scene with the same layout as the scenes shown in the experiment (Figure 10), presented using the real-scene box.

In Experiment 2 we adopted an optimised approach to data collection, allowing the experiment to be completed over a more practical duration per observer. Rather than comparing all possible pairs, Experiment 2 instead used an adaptive (active sampling) procedure in which pairs of conditions were selected to maximize the information gain on each comparison (where information gain is defined as the Kullback–Leibler divergence between the prior and posterior distributions (Mikhailiuk, Wilmot, Perez-Ortiz, Yue, & Mantiuk, 2021). As a result, the comparisons with obvious outcomes (e.g., between two very different conditions) that have little influence on the estimates are avoided. The adaptive procedure used (ASAP^2^; Mikhailiuk et al., 2021) ensures that each condition is compared with another at least once before the next batch of comparisons (the minimum-spanning-three approach; Li, Mantiuk, Wang, Ling, & Le Callet, 2018). The procedure utilizes information gathered from all previous batches/repetitions, including those from other observers. ASAP has been shown to require fewer comparisons to achieve the same estimation error as sorting methods (e.g., Swiss Chess System) and much fewer than when all combinations of pairs are compared (Mikhailiuk et al., 2021).

The experiment was conducted in three blocks:

• Block 1: depth sep. 0.4 D, 100 cd/m^2^ mean luminance
• Block 2: depth sep. 0.4 D, 1 cd/m^2^ mean luminance
• Block 3: depth sep. 0.6 D, 100 cd/m^2^ mean luminance

The mean luminance was calculated across the pixels belonging to an object (without the black background). Blocks 1 and 2 were completed in a single session, while Block 3 required a separate session on a different day as the display had to be reconfigured to a different depth separation. Each observer completed 70 comparisons per block, and each condition was compared with another at least 10 times by each observer. Due to attrition, three observers did not complete all three blocks. Two observers completed only the two 0.4 D blocks, and one observer completed only the 0.6 D, 100 cd/m^2^ block.

### Results

The active sampling approach used in Experiment 2 (Mikhailiuk et al., 2021) results in fewer trials per observer, per comparison, than in Experiment 1, and necessitates a different approach to computing Thurstone scale values (Thurstone, 1927), and to subsequent statistical analyses. Instead of calculating scale values for each observer (and averaging to produce overall results), we calculated single scale values per condition across all observers’ data within each luminance/depth-separation block. Note that as well as factoring in between-observer variance this approach also factors-in within-observer variance. Confidence intervals (95%) were obtained for each data point by bootstrapping (10,000 bootstrap samples). Statistical significance was then assessed, within each luminance/depth-separation block, as follows. We first determined that the bootstrapped samples were normally distributed (Kolmogorov–Smirnov test, α = 0.05). We then used two-tailed z-tests (Holm–Bonferroni corrected for six pairwise comparisons) to determine the statistical significance of differences between the conventional stereo baseline and each of the other six conditions. Because a pairwise-comparison experiment measures the differences between pairs of conditions, bootstrapping gives distributions of means that are correlated (i.e., that have non-zero elements in the covariance matrix). Our *z*-tests accounted for such covariance. One consequence of this is that the error bars shown in the plots visualize the distribution of potential outcomes (if the experiment is repeated), but they cannot be used to judge the outcome of the statistical test (as the statistical test is calculated on the difference of two correlated random variables; refer to 7.2 in Perez-Ortiz and Mantiuk (2017) for further discussion).

**Figure 11.**
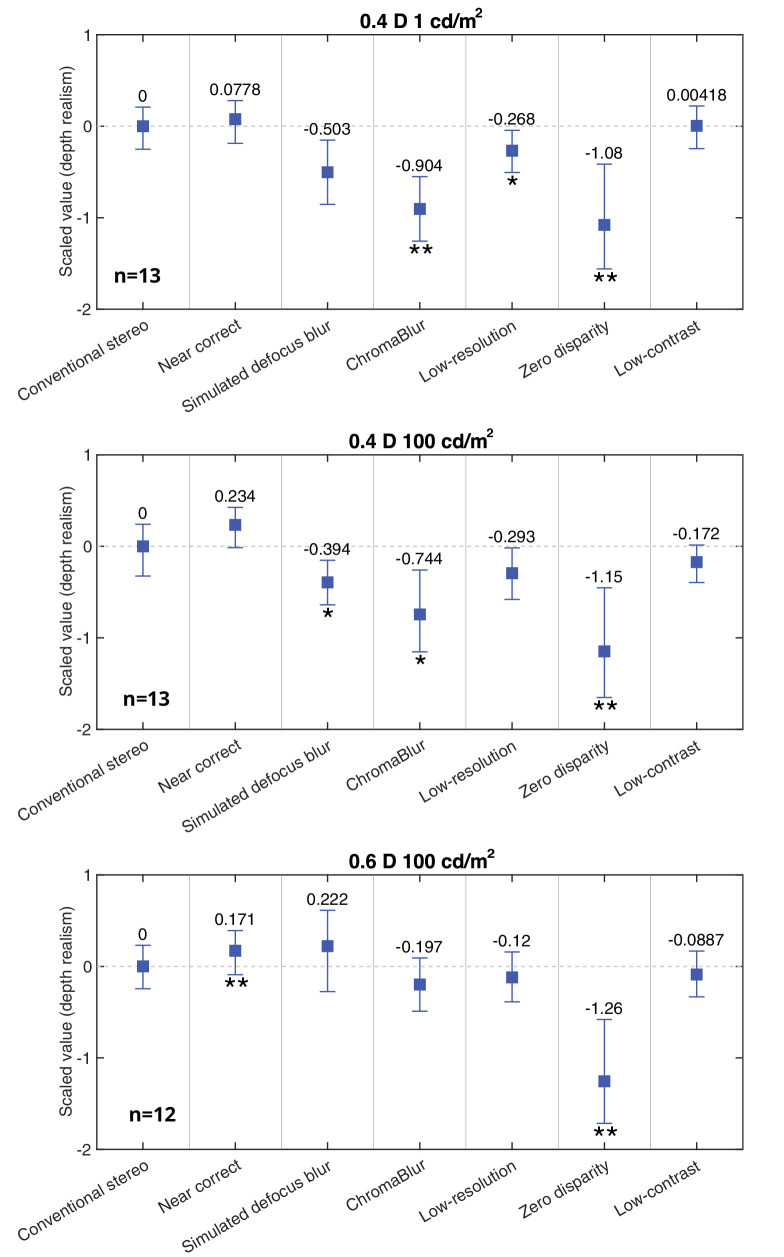
Experiment 2 results. Scale values for depth realism judgments in each experiment block. Lower values indicate lower realism. The relative scale values are shifted so that the conventional stereo baseline is at 0. Error bars denote 95% confidence intervals (estimated by bootstrapping). The * or ** symbols indicate a statistically significant difference from the baseline at *p* < 0.05 and *p* < 0.01, respectively (Holm–Bonferroni corrected for multiple comparisons; see main text for details). Note that the error bars do not indicate the outcome of the statistical test between a condition and the baseline (as explained in the main text).

Figure 11 plots the results separately for each luminance/depth-separation block of the experiment. Individual data can be found in the Appendix. (Tables of pairwise comparisons, showing the percentage of trials, aggregated across observers, on which each condition was selected as having more depth realism than every other condition can be found in the supplementary material on our project webpage^3^.) In all three blocks, depth realism was higher in the near-correct condition than for conventional stereo, but the effect was less pronounced than in Experiment 1, with a significant difference only at the larger depth separation (0.6 D, 100 cd/m^2^). Reproducing our previous work (March et al., 2022), we found no evidence that either method of rendering blur increases realism of depth separations. Indeed, if anything it results in a *reduction*, with significantly lower depth realism (compared to Conventional stereo) found with simulated defocus blur in the 0.4 D 100 cd/m^2^ block, and with ChromaBlur in both 0.4 D depth separation blocks. There was, however, no significant reduction in realism for either rendered blur condition at the larger depth separation (0.6 D, 100 cd/m^2^). Several observers reported that they where unable to fuse the out-of-focus object at this depth separation, which could explain the lower sensitivity to rendered blur in this block (see *General discussion*). Degrading quality by using 2-D presentation (zero disparity condition), as expected, resulted in substantial, and statistically significant, reduction in depth realism in all three blocks. As discussed earlier, it serves as a useful rationality check that perception of depth realism is reduced with 2-D presentation. The low contrast condition did not introduce a measurable drop in realism. There was a small but significant drop in realism due to low resolution, however, for the block with lower mean luminance (0.4 D, 1 cd/m^2^).

Taken together with the results of Experiment 1, these results suggest that (near) correct focus cues confer a less reliable increase in depth realism in more realistic imagery. Moreover, existing approaches to rendering defocus blur, including approximating effects of chromatic aberration, do not appear to increase depth realism for stimuli presented on a single, fixed focal plane. Next we turn to varifocal approaches to presenting near-correct focus cues.

## Experiment 3: Varifocal

Experiment 3 examined the efficacy for depth realism of another class of stereo 3-D displays that attempt to approximate correct focus cues—varifocal displays. In this approach, images are displayed on a single focal plane and the focal distance of the plane (and therefore of the whole scene) is adjusted contingent on eye-gaze to match the depth of the currently fixated scene point. In its simplest form this approach presents all image points sharply, independent of relative distance (i.e., it resembles a conventional stereo 3-D display that moves to different focal distances). The approach can also be combined with rendered blur, however, to provide a closer approximation to real-world focus cues (Cholewiak et al., 2017; see *Introduction: Display requirements for correct focus cues*). Focal distance to the display is typically varied using adaptive optics or by mechanically moving the display. In this experiment, we again presented objects at two distances, and used the two focal planes of the display to simulate the operation of a varifocal display. We tested whether rendered blur (“simple” simulated defocus blur and ChromaBlur) can improve depth realism in depicted scenes when combined with varifocal presentation. We compared these approaches to both conventional stereo 3-D and near-correct focus cues. As well as providing relevant baselines for varifocal performance, this also provided a replication of the near-correct vs conventional 3-D comparison in Experiment 2.

### Methods

Experiment 3 used the same apparatus and procedures as Experiment 2 (0.4 D depth separation, 100 cd/m^2^ luminance), but with different focus-cue conditions. The differences are described.

#### Observers

Eight observers (6 males, 2 females) aged 20 to 30 years took part in Experiment 3, two of whom also participated in Experiment 2.

#### Stimuli

In this experiment, we compared the following focus-cue conditions.

**Figure 12.**
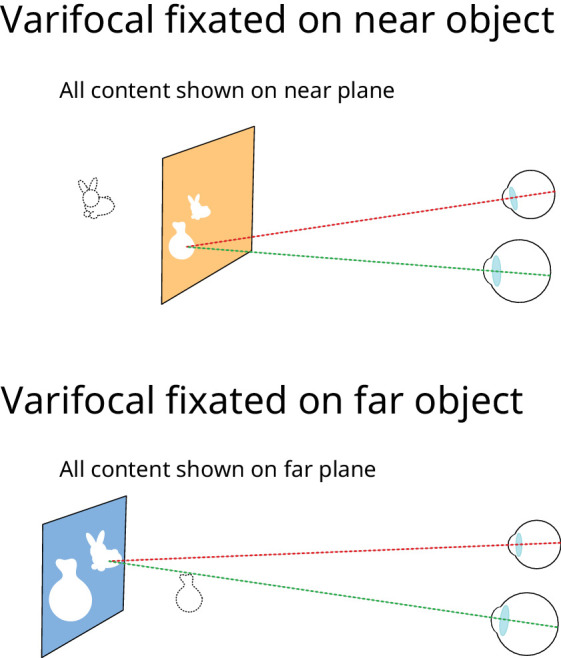
Varifocal presentation using two focal planes. Stimuli were presented in this way in Varifocal stereo, Varifocal simulated defocus blur and Varifocal ChromaBlur conditions. Near correct and Conventional stereo conditions were the same as in Experiment 2.

• Conventional stereo. As in Experiment 2.
• Near correct. As in Experiment 2.
• Varifocal stereo. Near and far objects presented on one focal plane, near or far, depending on which object was currently fixated, as illustrated in Figure 12. No rendered blur was applied.
• Varifocal simulated defocus blur. As varifocal stereo, but simulated defocus blur was applied as per Experiment 2.
• Varifocal ChromaBlur. As with varifocal stereo, but ChromaBlur rendered blur was applied to simulate defocus blur and chromatic aberration.

**Figure 13.**
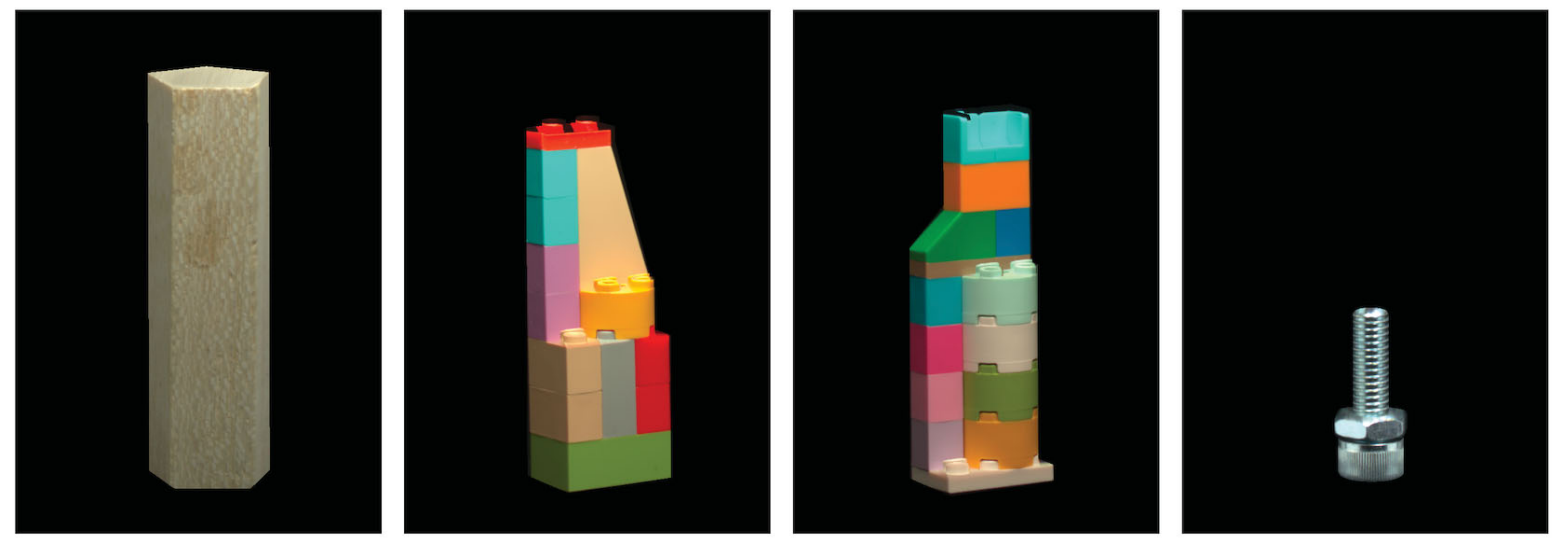
Renderings of the objects used to construct scenes displayed in Experiment 3.

The objects used to construct the scenes used in Experiment 3 are shown in Figure 13. The objects were slightly narrower, allowing smaller horizontal spacing than in Experiment  2 (5.37° vs. 6.6°), while still avoiding occlusions in either eye’s view.

The same eye-tracking procedure was used as in Experiment 2 to determine which object was currently fixated. Because switching between focal planes could result in a noticeable flicker (owing to small geometric or color differences between the far and near displays), which would appear unrealistic, the display was blanked for 200 ms when gaze was shifted from one object to another. This blank was present in all conditions, including near correct and conventional stereo (where there was no change in the focal planes used), so that the presence of a blank was not confounded with focus-cue condition. Thus, in the varifocal conditions fixating the near or far object determined which focal plane both objects were presented on, whereas in the near correct and conventional stereo conditions fixation was used only to trigger the blank period.

### Results

**Figure 14.**
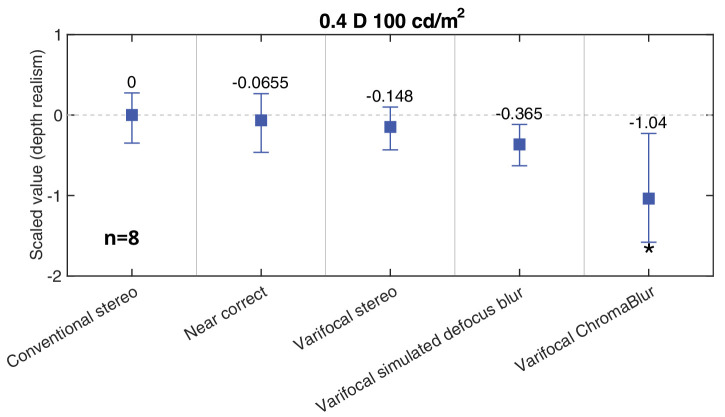
Experiment 3 results. Depth realism scale values for varifocal conditions, compared with conventional stereo and near correct (all displayed with a blank when switching gaze between the objects). The notation is the same as in Figure 11.

The results of Experiment 3 are shown in Figure 14. Again, individual data are shown in the Appendix (and percentage selections for each pairwise comparison can be found in the supplementary material). The data analysis methods and parameters thereof were the same as in Experiment 2. The results show no evidence that combining varifocal presentation with rendered blur improved depth realism compared to Conventional stereo. Only the ChromaBlur condition differed significantly from Conventional stereo and, as in Experiment 2 (with non-varifocal presentation), ChromaBlur caused a reduction in depth realism. As per the equivalent block in Experiment 2 (0.4 D depth separation, 100 cd/m^2^ luminance) there was again no significant improvement in depth realism in the near correct condition (the value was in fact marginally lower than for Conventional stereo, but not significantly so).

## General discussion

### Summary of results

Presenting correct focus cues in stereo 3-D imagery might be expected to enhance perceived realism of 3-D structure (depth realism). To examine whether this is the case, we compared depth realism judgments with conventional stereo 3-D stereo presentation, in which focus cues are incorrect, and when focus cues were presented (nearly) correctly, via multiple-focal-planes displays. We examined the effect of (near) correct focus cues both with reduced-cue stimuli (random-dot stereograms), where the role of focus cues might be expected to be most salient, and with relatively realistic stimuli more representative of practical applications of 3-D displays. We also explored whether various rendering-based approaches to simulating correct focus cues increased depth realism. Experiment 1 showed that correct focus resulted in systematically increased depth realism in reduced-cue scenes, indicating that focus cues do play a role in this aspect of the appearance of 3-D scenes. Experiments 2 and 3 found only limited evidence for benefits of correct focus cues, suggesting they may be less important for depth realism in more complex and realistic imagery. There are important caveats to this conclusion, however, which we explore below. We found that rendering-based approaches to approximating correct focus cues, if anything, reduced depth realism compared to conventional 3-D presentation. These findings highlight the wider challenges of presenting especially second-order aspects of focus cues correctly in 3-D imagery.

### Role of focus cues in depth realism

The results of Experiment 1 provide proof-of-principle evidence that depth realism can be increased by presenting correct focus cues. This is consistent with previous work showing that depth-dependent blur can generate a sense of “realness” to perceived 3-D structure similar to that derived from binocular stereopsis and motion parallax (Vishwanath & Hibbard, 2013), and demonstrates that at least under some circumstances correct focus cues confer benefits for perceptual realism in 3-D imagery. This finding also adds to accumulating evidence that retinal blur in general plays a more important role in perception than has often been thought (Marshall et al., 1996; Mather, 1997; Nguyen et al., 2005; Held et al., 2010; Vishwanath & Blaser, 2010; Held et al., 2012; Vishwanath & Hibbard, 2013; March et al., 2022).

Taken at face value, the limited benefits of near-correct focus cues in Experiments 2 and 3 suggest that presenting focus cues correctly is less important in relatively realistic scenes more typical of practical uses of 3-D imagery. Caution is needed in generalizing from Experiments 2 and 3 to applications of 3-D imagery in general, however, for two related reasons.

First, specific aspects of our stimuli may have reduced the reliability of signals from focus cues in Experiments 2 and 3, resulting in an underestimate of their role compared to naturalistic scenes. The objects were quite widely spaced (6.6° and 5.37° horizontal separation in Experiments 2 and 3, respectively) and so the defocused object was seen in parafoveal vision (and possibly diplopic with 0.6 D depth separation), reducing sensitivity to blur. Also, focus cues were slightly incorrect in Experiments 2 and 3, owing to the objects having depth relief yet being presented on a single focal plane, which may have reduced their effectiveness. Although the errors this introduced were small (<0.15 D) we cannot rule out this possibility. Moreover, to avoid confounding the role of focus cues with implementation-specific errors in their presentation, we intentionally avoided presenting several scene features that are likely to provide reliable signals from focus cues in natural scenes, but that multiple-focal-planes displays cannot present accurately. This includes occlusions, transparency, and surfaces that extend in depth between focal planes (the ground plane, and large object surfaces, for example) (MacKenzie et al., 2010; Ravikumar et al., 2011; Narain et al., 2015).

Second, and more generally, the difference between effects of (near) correct focus in reduced-cue (Experiment 1) and more realistic scenes (Experiments 2 and 3) show that the contribution of focus cues to depth realism is not fixed, but instead depends on scene content. This finding is consistent with depth realism conforming to the logic of depth-cue integration, in which all available cues are used, and their contributions depend on their relative reliabilities in a given situation (e.g. Knill & Saunders, 2003; Hillis et al., 2004). If depth realism behaves similarly, focus cues will contribute to depth realism in naturalistic imagery in some situations (in which they are particularly informative). Work on depth perception suggests this contribution is difficult to anticipate, because the relative reliabilities of individual cues vary substantially, not only across different scenes, but also within the same scene (Knill & Saunders, 2003; Hillis et al., 2004; Watt et al., 2005a; Held et al., 2012), and even for different regions of a single object or surface (Hillis et al., 2004).

The above arguments, taken together with the proof-of-principle findings of Experiment 1, suggest it is likely that focus cues will contribute to depth realism in some circumstances encountered in naturalistic imagery. General 3-D display solutions should therefore ideally present focus cues correctly. It is interesting to note, however, that demonstrating this unambiguously requires presenting focus cues fully correctly, in truly naturalistic scenes that contain continuous, extended surfaces, and occlusions, for example. Presenting second-order aspects of blur fully correctly, including dynamic variations that depend on accommodative state, is not achievable with current display technologies (see *Display requirements for correct focus cues*).

Contrary to our expectations we did not observe a meaningful difference in observer perceptions of depth realism when varying luminance (in our realistic-object experiments). Pupil diameter increases when luminance decreases, which should result in a smaller depth of focus, and therefore more retinal blur, in the low luminance session for the near correct condition. It could be that the lower contrast sensitivity at low luminance (Van Nes & Bouman, 1967) made the differences in blur more difficult to detect. It is also possible that observers are accustomed to variations in blur due to changes in pupil size from real-world viewing, and can take them into account, such that increased but still correct amounts of blur do not result in a larger effect on depth realism (see also *Experiment 1: Discussion*).

We also did not find evidence of a systematic relationship between the depth separation between objects and the effect of (near) correct focus cues on depth realism. In Experiment 2, we found a statistically significant improvement owing to near-correct focus cues (vs. conventional stereo) at 0.6 D, but not at 0.4 D (at the same luminance; Figure 11). In Experiment 1, however, in which depth separation was systematically varied, the effect of correct focus cues on depth realism was independent of depth separation (Figure 4B).

### Can demand characteristics explain our results?

Judgments of subjective qualities such as depth realism are potentially vulnerable to experiment demand characteristics (Orne, 1962). Here, the principal concern is that observers might be aware that focus cues were the object of our investigation, and infer that they should base their judgments on the presence of blur in the retinal image, rather than the evoked sense of depth realism per se. We were most concerned about this for the sparse stimuli used in Experiment 1, in which blur (in the non-fixated stimulus plane; Correct focus cues condition) was most salient, and we included a foil condition (unrealistic Gaussian blur) to identify (and remove) observers who may have responded in this way. We chose not to include a similar foil in Experiments 2 and 3 because blur was far less salient for the realistic images used in these experiments (especially with 0.4 D depth separation), and so the experimental manipulation was less likely to be explicitly noticed (a foil may even have drawn attention to the subtle experimental manipulations; see Figure 8). This means we cannot definitively rule out that the results of Experiments 2 and 3 were affected by demand characteristics relating to presence of blur. Our data suggest this is unlikely, however. Judgments based purely on blur would be expected to result in higher depth realism values for all three conditions in which the non-fixated object appeared blurred, independent of whether this was caused by the eye’s optics (near correct condition) or by rendering (Simulated defocus blur and ChromaBlur conditions). This result is not what we found. Overall, we found a dissociation between the effects of natural and rendered blur. And only one individual observer consistently judged these three conditions as having higher depth realism (observer 12 in Experiment 2; see Appendix), and so *could* have responded on the basis of detecting blur (although they could of course have genuinely experienced higher depth realism in these conditions). Removing this observer from the Experiment 2 analysis (as a precautionary measure) did not substantially alter our findings, although the reduction in depth realism with rendered blur became more reliable (see supplementary material). We, therefore, consider it unlikely that our principal findings can be explained by demand characteristics causing depth realism judgments to be based on presence of blur rather than depth realism.

### Individual differences

As discussed previously, there were individual differences in the effect of correct focus cues in Experiment 1, suggesting that the contribution of focus cues to perceptual realism varies across individuals (see Appendix). The individual data for Experiment 2 were arguably more consistent, with most observers showing clear evidence of reduced realism in the rendered blur conditions compared to conventional stereo presentation (see Appendix). In Experiment 3, with varifocal presentation, two (of eight) observers showed evidence of higher depth-realism judgments in the rendered blur conditions, again suggesting the contribution of focus to perceptual realism varies across individuals (though the reduction in depth realism with Chromablur was large and consistent in the remaining observers). It is possible that our aberration (blur) model better matched these two observers’ own optics (as discussed elsewhere in this article). Note, however, that inferences from individual data from Experiments 2 and 3 must be treated with caution: the adaptive testing protocol optimized for the best estimates across the population sample rather than for individual observers, and the exact number trials (and the pairwise comparisons) each data point is based on varies (although each estimate is based on at least 10 comparisons).

### Implications for gaze-contingent rendering of focus cues

We found essentially no evidence that simulating blur via rendering-based approaches (depth-of-field rendering) improved depth realism. ChromaBlur, in particular, resulted in significantly decreased depth realism in most cases, including when coupled with gaze-contingent changes in the focal distance to fixation in Experiment 3 (varifocal presentation). These findings are consistent with previous work showing that rendered defocus blur is also less effective as a depth cue than natural blur (Held et al., 2012; Langer & Siciliano, 2015). They also suggest that observers are sensitive to second-order blur cues, consistent with previously demonstrated sensitivity of depth perception and accommodation control to second-order aspects of blur (Fincham, 1951; Campbell & Westheimer, 1959; Charman & Tucker, 1978; Kotulak & Schor, 1986; Kruger et al., 1993; Aggarwala et al., 1995; Lee et al., 1999; Artal et al., 2004; Fernández & Artal, 2005; Nguyen et al., 2005; Chen et al., 2006; Chin et al., 2009; MacKenzie et al., 2010; Sawides et al., 2012; Sebastian et al., 2015; Cholewiak et al., 2017).

Presenting second-order blur cues correctly via rendering is inherently difficult. In both our Simulated defocus blur and ChromaBlur conditions rendered blur did not take into account individual eye optics, and imagery may look more or less realistic depending on how well the aberration (blur) model matches the characteristic of the observer’s eye (Artal et al., 2004; Maiello, Chessa, Solari, & Bex, 2015). While it is conceivable to measure the user’s eye optics in specialist applications (and model them in creating rendered blur), this is not currently practical in most settings. Also, rendered blur does not reproduce depth-dependent effects of accommodation microfluctuations correctly, even with varifocal presentation. This may also reduce depth realism. Although these effects could also be simulated, it would require high-precision real-time measurement of the accommodative state of a moving eye, which is currently not practical.

The fact that ChromaBlur, specifically, performed poorly for judgments of depth realism may seem surprising given previous findings that it made scenes appear more realistic (Cholewiak et al., 2017). Our result replicates our previous finding that ChromaBlur causes a reduction in depth realism compared both to conventional stereo, and near-correct focus cues (March et al., 2022). This apparent discrepancy could reflect differences between the stimuli used by us and by Cholewiak et al. (2017): they used monocular presentation, and their stimuli depicted more complex, achromatic scenes. There are also several factors that may limit the capability of ChromaBlur to faithfully reproduce the appearance of chromatic aberrations more generally. First, the method assumes the eye is focused at a single wavelength (Cholewiak et al., 2017) and if this value is incorrect it would introduce subtle chromatic inaccuracies in the displayed image (Thibos, Ye, Zhang, & Bradley, 1992). Second, displays do not reproduce the entire spectra of natural light, but instead use three color primaries. The color fringes are calculated by altering the blur radius applied to each primary, which could result in simulated chromatic aberration that differs from that encountered in real-world viewing. Third, the required deconvolution step (to find a display image that would produce the intended retinal image) generates an impossible image with negative color values, which need to be clipped before being shown on the display. This, too, could introduce inaccuracies into the displayed image. ChromaBlur may still be advantageous in varifocal displays because it can stimulate an accommodation response in an appropriate direction (Cholewiak et al., 2017). Our data suggest it could negatively affect the appearance of the resulting images, however.

It is also difficult to present realistic transitions in defocus blur, and focal distance (for varifocal presentation), in gaze-contingent depth-of-field rendering. In our varifocal conditions (Experiment 3), the blanks introduced during changes in fixation may have made the stimuli look less realistic than an equivalent real scene. This control was necessary because achieving a perfect match between stimuli presented on different focal planes is difficult and any inaccuracies in this would likely bias judgments of depth realism toward those conditions that did not dynamically change focal plane (e.g Conventional stereo). Similarly, we could not accurately model dynamic effects of eye accommodation on blur when the gaze was shifted between objects (Experiments 2 and 3), and merely simulated a smooth transition that may not have matched natural accommodation-driven changes. The same challenges are present when implementing gaze-contingent rendering of focus cues and varifocal presentation in practical settings (where blanking is clearly not appropriate). For example, it may be necessary to generate dynamic transitions in blur as accommodation adjusts. Those transitions may need to be subtly different to match the natural patterns of different observers (Sun et al., 1988).

It is also interesting here to evaluate the contribution of rendered blur in relation to other display attributes when attempting to achieve perceptual realism. In Experiment 2, as expected, Zero disparity presentation caused a large, clearly significant reduction in depth realism in all luminance/depth-separation conditions tested. Reducing contrast had no effects, which might be expected since contrast is not directly a cue to depth. Halving display resolution (albeit from a high initial value) resulted in a significant reduction in just one of three luminance/depth-separation conditions (0.4 D at 1 cd/m^2^). It seems therefore that the rendered blur manipulations, and ChromaBlur in particular, resulted in a more consistent reduction in depth realism than other explicit ways of degrading image quality, with the exception of Zero disparity (i.e., conventional 2-D) presentation.

These factors highlight some of the challenges inherent in presenting focus cues correctly using gaze-contingent depth-of-field rendering, and varifocal presentation, due to dependence on rendering for creating fine-scale aspects of blur. Our data suggest that these approaches can create worse appearance than conventional stereo presentation, at least when based on a generic model of eye optics. Performance may also be improved by addressing transitions in blur and focal distance more effectively.

### Implications for display development

A general display solution, for use in Virtual Reality applications for example, would ideally present 3-D imagery that is perceptually compelling, highly realistic, and free from adverse user issues, across the whole range of naturally encountered viewing distances. It is already established that achieving this requires a solution to coarse errors in focus-cue presentation (caused by large mismatches between the focal distance of the display and image points in the depicted scene), in order to keep vergence–accommodation conflicts within tolerable levels (Hoffman et al., 2008; Shibata et al., 2011). The current findings demonstrate how incorrect focus cues can also cause unwanted effects on the appearance of 3-D imagery, however, by reducing perceptual realism. Moreover, these effects are evident even in physically small scenes (as tested here), in which the mismatch between vergence and accommodation (focal) distances is small. That is, conventional 3-D presentation can cause measurable reductions in depth realism for stimuli in which effects of vergence–accommodation conflict per se would largely be inconsequential (within the recommended zone-of-comfort for 3-D content derived by Shibata et al., 2011, for example). This finding suggests that future 3-D displays should aim to present not only coarse aspects of focus cues, but also second-order aspects of blur, if perceptual realism is to be maximised. We speculate that sensitivity to these perceptual aspects of incorrect focus cues may become increasingly evident as displayed imagery approaches being indiscriminable from viewing the real world (Zhong et al., 2021).

Display requirements for stimulating accurate accommodation, and thereby eliminating vergence-accommodation conflicts, are less demanding than for presenting second-order blur cues and existing approaches have the potential to solve this problem with sufficient development. Multiple-focal-planes displays, combined with image-interpolation techniques, have been shown to stimulate accurate, continuously variable accommodation responses to distances between focal planes (MacKenzie et al., 2010; MacKenzie et al., 2012). And varifocal presentation inherently removes vergence-accommodation conflicts, by matching the focal distance of the display to the simulated distance of the fixated point. It should be noted, however, that varifocal presentation requires very accurate eye-tracking if it is to be fully effective. Complex, realistic 3-D scenes often contain steep depth gradients (caused by occlusion edges or transparency, for example) and so even small errors in eye-tracking could cause large errors in focal-distance positioning of the display. Moreover, in their current forms neither approach results in fully correct second-order aspects of blur.

It seems reasonable to assume that the most challenging second-order blur signals to simulate are higher-order aberrations (which depend on individual eye optics) and effects of accommodation microfluctuations (which are difficult to measure). It seems likely therefore that presenting second-order blur signals correctly in 3-D displays might best be achieved by approaches that leverage the eye’s own optics to create these signals. This includes holographic (Chakravarthula et al., 2022) and light field or tensor displays (Huang et al., 2015), although these currently have limited spatial resolution, as described earlier. It also, in principle, includes multiple-focal-planes displays, provided the focal planes are very closely spaced. Typically there are strict limitations on the number of focal planes, and therefore the inter-plane spacing that is practical with mirror/beamsplitter and time-multiplexed approaches (MacKenzie et al., 2010; though see (Chang et al., 2018). Some applications may require only a small range of depths to be presented, however, potentially allowing the same number of focal planes to be spaced more closely. Also, novel approaches continue to be developed, including a recent study that used a locally addressable phase modulator to create different focal distances in different spatial regions of a conventional display, with reasonably high resolution in focal depth (Qin, Chen, O'Toole, & Sankaranarayanan, 2023). Note, however, this approach is not strictly a multiple-focal-planes display, in that it cannot present multiple focal distances along a single line of sight (Akeley et al., 2004).

## Conclusions

We found proof-of-principle evidence that correct focus cues can improve the sense of perceptual realism of 3-D scene structure—depth realism—in stereo 3-D imagery. Although these effects were less clear in more realistic imagery, our results nonetheless suggest that the role of focus cues is scene-dependent. This implies that focus cues are likely to contribute under some circumstances, which may be difficult to predict. We found that gaze-contingent rendering of focus cues diminished depth realism, providing evidence that second-order aspects of retinal blur may need to be presented correctly if realism is to be maximized. Inherent challenges in presenting these signals correctly via rendering suggest that approaches that leverage the eye’s optics to create blur signals, even though they are more technically challenging, may provide a more effective path towards highly realistic 3-D imagery.

## Figures and Tables

**Figure A1.**
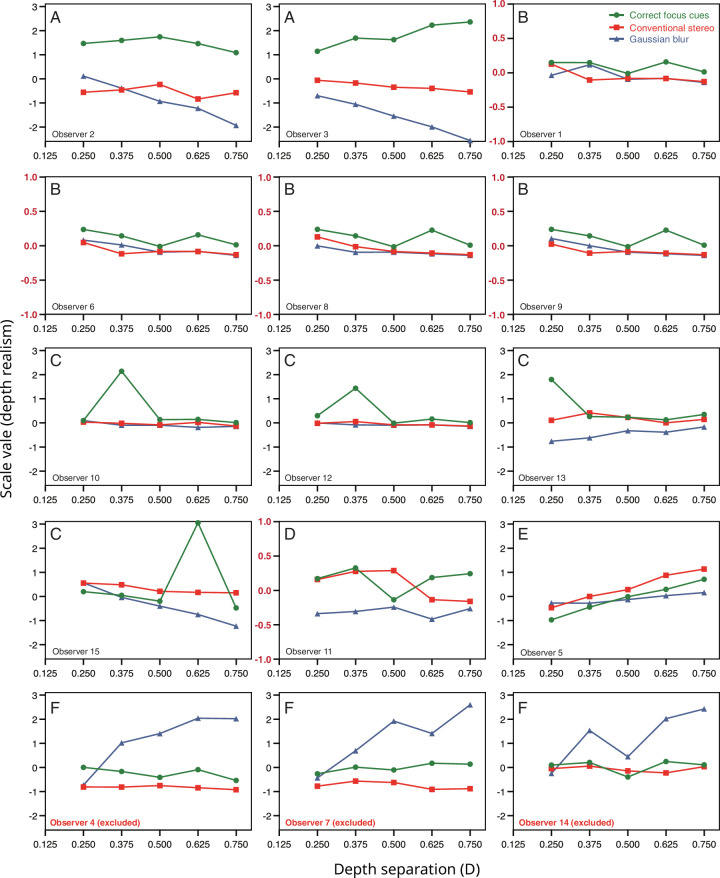
Depth realism judgments for individual observers in Experiment 1. Each observer’s Thurstonian scale values are plotted as a function of depth separation for each focus-cue condition. The plots are categorised by the trends in the data. (A) Clear benefit of correct focus cues over conventional stereo. (B) Small but consistent benefit of correct focus cues (note smaller y-axis range denoted by red values). (C) Clear benefit of correct focus cues at one depth separation. (D) No consistent pattern. (E) Consistent benefit for conventional stereo over correct focus cues. (F) Observers who consistently selected rendered Gaussian blur stimuli as having more depth realism than the other conditions, and were excluded from the analysis.

**Figure A2.**
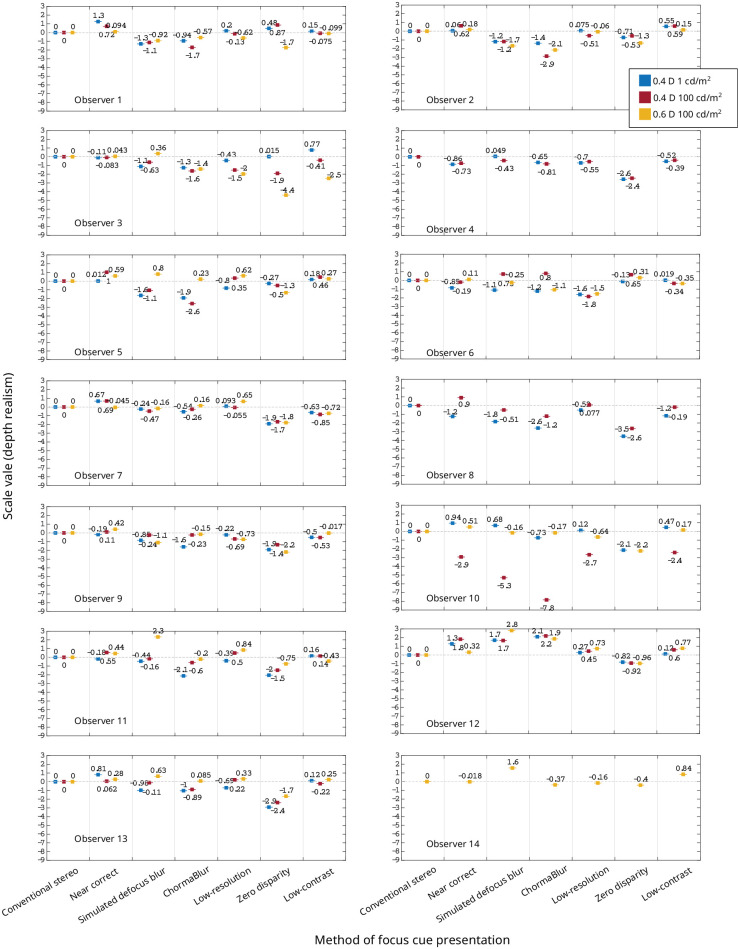
Individual results for Experiment 2. Here, the MLE scaling was performed separately for each participant rather than on aggregated data (see main text). The colors correspond to the three blocks of the experiment, each with different depth separation and mean luminance. Three observers (4, 8, and 14) did not complete all three blocks of the experiment.

**Figure A3.**
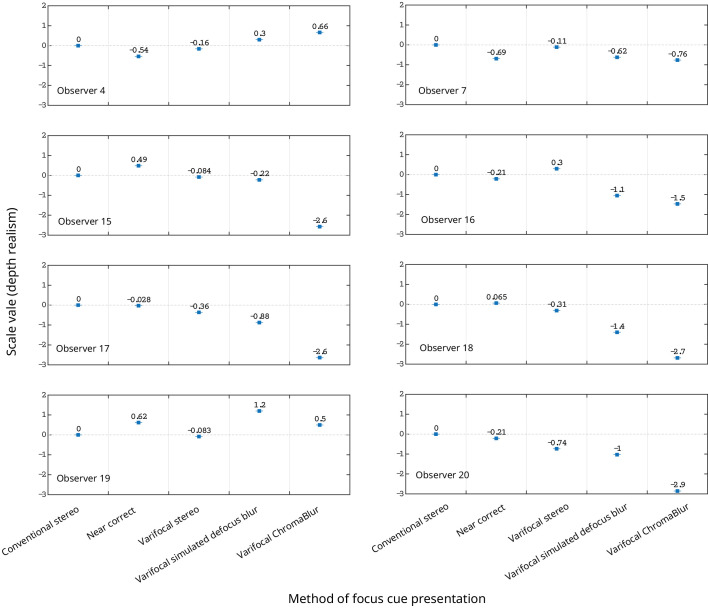
Individual results for Experiment 3 (varifocal presentation), plotted in the same format as Figure 14. Again, MLE scaling was performed separately here for each participant rather than on aggregated data (see main text). Note, observers 4 and 7 also participated in Experiment 2.

## Footnotes

1
*pwcmp* software: https://github.com/mantiuk/pwcmp.

2 The code for the ASAP active sampling method can be found at https://github.com/gfxdisp/asap.

3
https://www.cl.cam.ac.uk/research/rainbow/projects/focus_cues/.
